# The Motility of a Human Parasite, *Toxoplasma gondii*, Is Regulated by a Novel Lysine Methyltransferase

**DOI:** 10.1371/journal.ppat.1002201

**Published:** 2011-09-01

**Authors:** Aoife T. Heaslip, Manami Nishi, Barry Stein, Ke Hu

**Affiliations:** Department of Biology, Indiana University, Bloomington, Indiana, United States of America; University of Michigan, United States of America

## Abstract

Protozoa in the phylum Apicomplexa are a large group of obligate intracellular parasites. *Toxoplasma gondii* and other apicomplexan parasites, such as *Plasmodium falciparum,* cause diseases by reiterating their lytic cycle, comprising host cell invasion, parasite replication, and parasite egress. The successful completion of the lytic cycle requires that the parasite senses changes in its environment and switches between the non-motile (for intracellular replication) and motile (for invasion and egress) states appropriately. Although the signaling pathway that regulates the motile state switch is critical to the pathogenesis of the diseases caused by these parasites, it is not well understood. Here we report a previously unknown mechanism of regulating the motility activation in *Toxoplasma*, mediated by a protein lysine methyltransferase, AKMT (for Apical complex lysine (K) methyltransferase). AKMT depletion greatly inhibits activation of motility, compromises parasite invasion and egress, and thus severely impairs the lytic cycle. Interestingly, AKMT redistributes from the apical complex to the parasite body rapidly in the presence of egress-stimulating signals that increase [Ca^2+^] in the parasite cytoplasm, suggesting that AKMT regulation of parasite motility might be accomplished by the precise temporal control of its localization in response to environmental changes.

## Author Summary

*Toxoplasma gondii* is one of the most successful human parasites, infecting ∼20% of the total world population. It is the most common cause of congenital neurological defects in humans, and an agent for devastating opportunistic infections in immunocompromised patients. To cause diseases, *Toxoplasma gondii* and other related parasites, such as *Plasmodium falciparum,* must reiterate their lytic cycle, comprising host cell infection, intracellular replication and parasite egress. At each step of the lytic cycle, the parasite tightly regulates its motility, being completely immotile while intracellular, and becoming highly motile as it leaves the host cell. Changes in local ionic conditions are known to trigger this rapid transition from immotile to motile. In this study, we report a previously unknown mechanism of regulating the motility activation in *Toxoplasma*, mediated by a novel protein lysine methyltransferase, AKMT (for Apical complex lysine (K) methyltransferase). The depletion of this protein greatly inhibits the parasite's ability to invade into and egress from the host cell due to impaired motility activation. Interestingly, the localization of AKMT in the parasite is sensitive to egress-stimulating signals, suggesting that AKMT regulation of parasite motility might be accomplished by the precise temporal control of its localization in response to environmental changes.

## Introduction

*Toxoplasma gondii* is one of the most successful human parasites, infecting ∼20% of the total world population. It belongs to the phylum Apicomplexa, which includes *Plasmodium falciparum,* the most lethal form of malaria parasites [1]. *T. gondii* is the most common cause of congenital neurological defects in humans, and an agent for devastating opportunistic infections in immunocompromised patients. Its pathogenesis absolutely depends on the parasite's ability to reiterate its lytic cycle, which is composed of host cell invasion, intracellular replication and egress.

**Figure 1:**
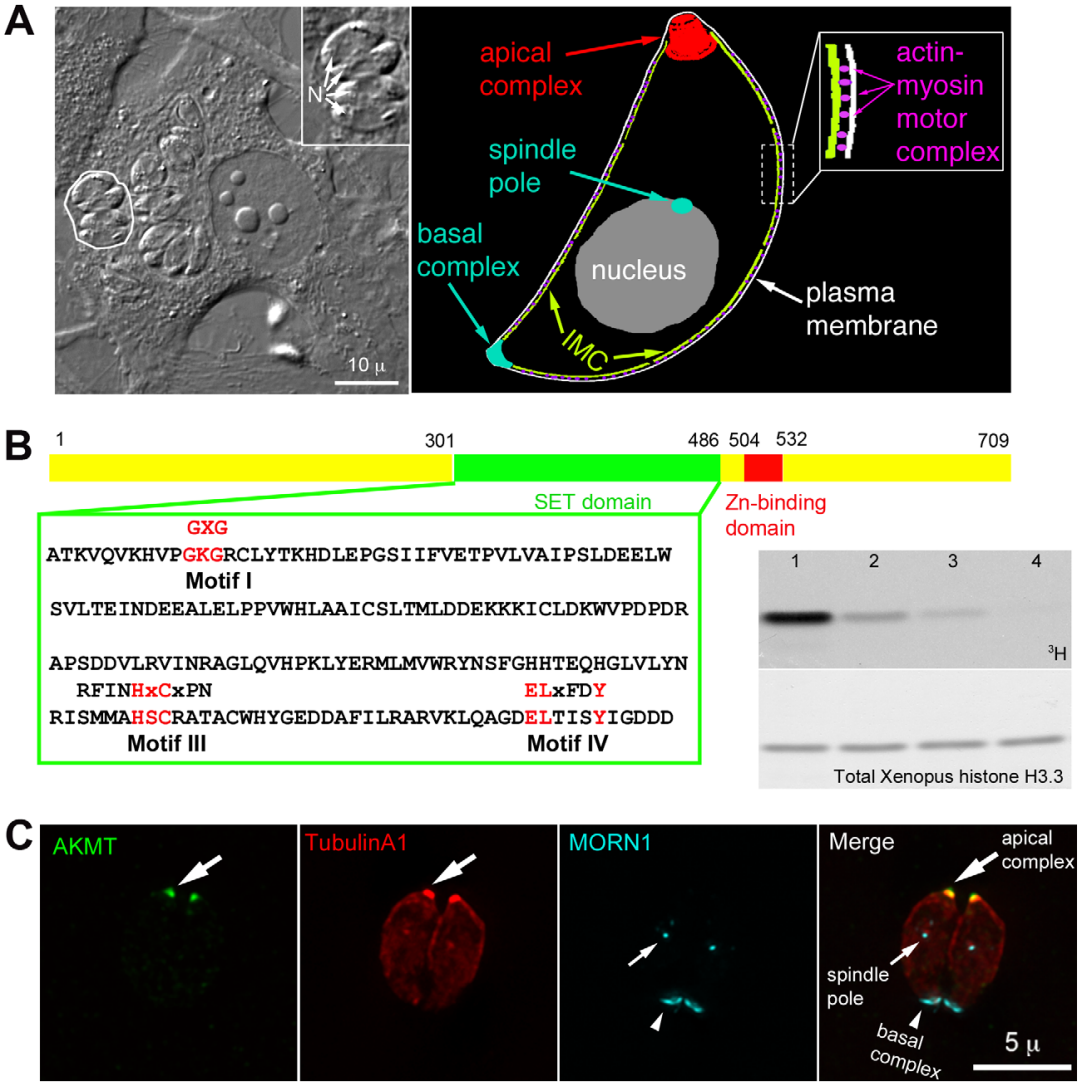
AKMT is a novel lysine methyltransferase localized to the apical complex in intracellular parasites. (A) *Left:* DIC image of human fibroblasts infected with *T. gondii.* The parasites live within a specialized parasitophorous vacuole, which is created from host cell plasma membrane during invasion. A parasitophorous vacuole containing 4 parasites (outline traced by a white border) is shown in the enlarged inset (N: nucleus). Inset: 1.5X magnification. *Right:* Cartoon drawing showing several membrane and cytoskeletal structures referred to in the text. For clarity, cortical microtubules of the parasite are not shown. Also not shown is the portion of the motility apparatus that mediates the interaction between the parasite and the host cell surface. (B) *Left:* AKMT contains SET (green) and zinc-binding (red) domains and is a predicted PKMT. The SET domain of canonical PKMTs contain four highly conserved motifs that are involved in different aspects of methyltransferase reactions: motif I for SAM binding; motif II for catalytic methyl transfer; motif III and IV for SAM binding and target lysine-binding. Three of the four motifs found in canonical PKMTs can be identified in AKMT, which contains a well-conserved motif I, and semi-conserved motifs III and IV. However, no conservation with the conventional motif II can be found in AKMT. Conserved amino acids in each motif found in canonical SET domains are shown above the *T. gondii* AKMT sequence. *Right*: Despite the unconventional SET domain, AKMT is a functional KMT enzyme. It can methylate an artificial substrate, Xenopus histone H3.3, *in vitro* in the presence of ^3^H-S-adenyl-L-methionine (^3^H-SAM). Top: ^3^H signal of histone in PKMT reactions containing ^3^H-SAM; 1 µg Xenopus histone H3.3; and recombinant FLAG-AKMT in the amount of 0.5 µg (lane1), 0.1 µg (lane 2), 0.05 µg (lane 3), 0.01 µg (lane 4). Bottom: The same blot stained with amido black to show total histone H3.3. Histone labeling can be detected when as low as 0.05 µg (lane 3) of FLAG-AKMT is present in the reaction. (C) Fluorescence images of two intracellular parasites at interphase showing that AKMT is localized to the apical complex (large arrows), a pattern observed in 100% of the hundreds of interphase parasites examined. *Green:* anti-AKMT; *red*: mCherryFP-TubulinA1 [43], [44] highlighting all the tubulin containing structure in the parasite, including the main body of the cytoskeletal apical complex (large arrows); *cyan*: eGFP-MORN1 [32], [43], [45] highlighting the basal complex of the parasite (arrowheads) as well as the spindle pole (small arrows). The host cell is not visible in these images, because it is not fluorescent.

During infection, *T. gondii* moves along the host cell surface using actomyosin-based gliding motility, attaches, and then rapidly invades, establishing a parasitophorous vacuole in which the parasite replicates [2]–[7] (Figure 1A). The invasion of the host cell is driven by parasite motility. Immediately after invasion, however, the parasite becomes non-motile to prevent premature rupture of the host cell, thus allowing for multiple rounds of replication within the same cell and optimal utilization of the host's resources. When the intracellular parasites sense unfavorable conditions, the non-motile parasites rapidly switch back to the motile state, actively disrupt the host cell, disseminate, and invade into new host cells. Therefore, success in completing the lytic cycle relies on the timely regulation of motility at each step in response to the changes in environmental conditions. It has been established that for intracellular parasites, the most important trigger for egress is the increase in [Ca^2+^] in the parasite cytoplasm, which is stimulated by the decrease in [K^+^] in host cell cytoplasm when the host cell plasma membrane is ruptured [8]. The Ca^2+^ signal then prompts several dramatic behavioral changes, including the extension of the cytoskeletal apical complex, a set of apicomplexan-unique cytoskeletal structures at the anterior end of the parasite (Figure 1A); the secretion from micronemes, a membrane-bound organelle; and the stimulation of parasite motility [2], [8]–[16].

The parasite motility is driven by a myosin motor complex containing 5 known components: an unconventional class XIV myosin (TgMyoA), a TgMyoA associated myosin light chain, and 3 other proteins, TgGAP40, TgGAP45 and TgGAP50, that are important for assembling and anchoring the complex to the inner membrane complex (IMC), a set of flattened vesicles underlying the parasite plasma membrane [14], [17]–[27] (Figure 1A). Actin filaments located between the plasma membrane and the IMC are anchored to the host cell surface by a number of linker proteins, including aldolase and a microneme protein MIC2, thereby translating the movement of myosin motors along the actin filaments into parasite movement [2], [11], [19], [20], [22], [24]–[27].

While the force generating apparatus of parasite motility has been well characterized, little is known about the signaling components that regulate motility. So far the only signaling components known to affect motility activation are calcium dependent protein kinase 1 (TgCDPK1) and cGMP dependent protein kinase (TgPKG), which regulate parasite motility through controlling the secretion of adhesive components of the motility apparatus (*e.g.* MIC2) from micronemes [28]–[31]. Here we report a novel protein lysine (K) methyltransferase (PKMT) that mediates a previously unknown mechanism of regulating the motility activation in *T. gondii.* This protein is concentrated in the apical complex of intracellular parasites, thus named Apical complex lysine (K) methyltransferase (AKMT). AKMT depletion significantly debilitates parasite motility activation, which compromises invasion and egress, thus severely impairs the lytic cycle. Interestingly, AKMT redistributes from the apical complex to the parasite body rapidly upon exposure to egress-stimulating signals, suggesting that parasite motility might be regulated by the precise temporal control of AKMT localization in response to ionic changes in the environment.

## Results

### AKMT is a novel lysine methyltransferase enzyme localized at the apical complex in intracellular parasites

TgAKMT (TGME49_016080, EupathDB) was first identified in a proteomic screen as a putative component of the cytoskeletal apical complex, an apicomplexan-specific structure located at the anterior end of *T. gondii* [32]. This protein was predicted to contain a SET (Ssu(var)3–9, Enhancer of zeste (E(z)), and trithorax (trx)) domain and a zinc binding domain, signatures of protein lysine(K) methyltransferases (PKMTs) (Figure 1B and S1A) [33], [34]. The SET domain of AKMT, however, is unusual, because it does not contain a conserved catalytic domain (motif II) shared by conventional PKMTs (Figure 1B). Orthologs of AKMT containing highly similar SET and zinc-binding domains (44–58% identity to the corresponding region in the AKMT) were found in the genomes of other apicomplexan parasites such as *Plasmodium*, *Cryptosporidium*, *Theileria and Babesia* (Figure S1A). We examined AKMT lysine methyltransferase activity *in vitro*, and found that recombinant AKMT protein can methylate an artificial substrate, Xenopus histone H3.3, in the presence of the cofactor S-adenosyl-L-methionine (SAM) (Figure 1B, right). Tandem mass spectrometry analysis of the methylated H3.3 detected two methylated lysine residues (Figure S1B). AKMT therefore is an active lysine methyltransferase. Although not visible in the autoradiograph of Figure 1B, we often see a weak band corresponding to AKMT self-methylation (*c.f.* Figure 7). Masspectrometry showed that K307 and K312 were methylated. We do not yet know if this actually is an *in vitro* artifact or has some physiological significance.

Unlike canonical histone lysine methyltransferases [35]–[42], AKMT was not detected in the nucleus, rather it was localized to the apical complex (highlighted by mCherryFP-TublinA1 [43], [44]) in interphase intracellular parasites as shown by labeling with a rat anti-AKMT antibody (Figure 1C). We further investigated AKMT localization at different stages of parasite replication. Immunofluorescence assays showed that the recruitment of AKMT to the daughter apical complex could be detected as early as the initial assembly of the apical and basal complexes (highlighted by eGFP-MORN1 [32], [43], [45])(Figure S1C) two cytoskeletal structures built at the beginning of daughter construction. Similar to the interphase parasite, AKMT was not detected in the nucleus of dividing parasites. Therefore, AKMT associates with the apical complex in intracellular parasites regardless of the stage of parasite replication.

### Creation of a Δ*akmt* parasite line using a Cre-LoxP technique

**Figure 2:**
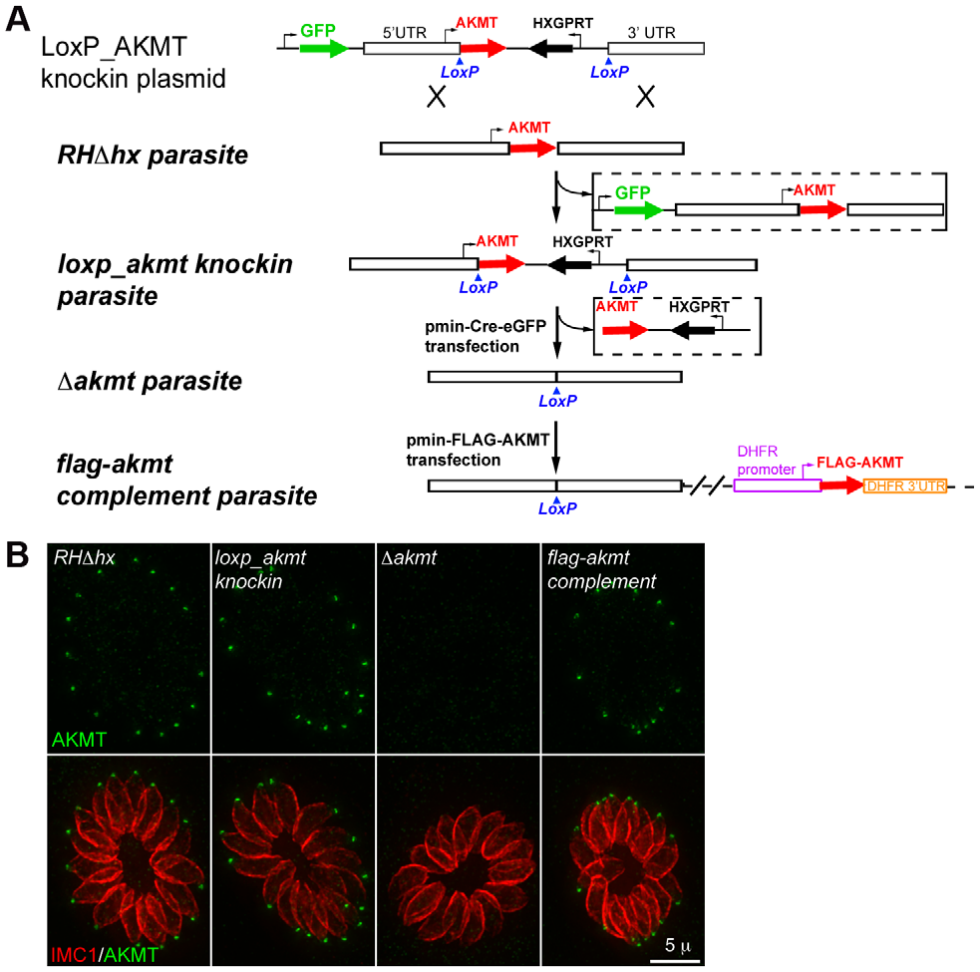
The generation and characterization of the AKMT knockout (*Δakmt*) mutant. (A) Procedure for generating the *Δakmt* parasite line. See text for details. (B) Immunofluoresence assay of intracellular *RHΔhx*, *loxp_akmt knockin*, *Δakmt* and *flag-akmt complement* parasites showing undetectable levels of AKMT in *Δakmt* parasites and the correct localization of FLAG-AKMT in the complemented parasites. *Green*: anti-AKMT; *red*: anti-IMC1. Each image shows 16 parasites contained within one parasitophorous vacuole. The host cells are not visible in these images, because they are not fluorescent.

To determine the function of AKMT in *T. gondii,* we first created AKMT knock-out (*Δakmt*) parasites using a Cre-LoxP based approach (Figure 2A) [46]–[48]. As the first step, we created a “*loxp_akmt knockin*” parasite line where the endogenous *AKMT* locus in the *RHΔhx* parasite was replaced with the AKMT coding sequence plus selectable marker HXGPRT, flanked by two LoxP sites. Replacement of the endogenous locus was confirmed by genomic PCR (Figure S2A). EGFP tagged Cre recombinase (Cre-eGFP) was then transiently expressed in the *loxp_akmt knockin* parasites, which excised the LoxP-flanked region, containing the AKMT coding sequence and HXGPRT expression cassette, from the genome. Two knock-out clones (*Δakmt-1* and *Δakmt-2*) obtained from two independent Cre-eGFP transfections were selected and the absence of AKMT was confirmed by immunofluorescence (Figure 2B), genomic PCR, and Western blot (Figure S2). The knock-out parasites were then complemented with FLAG-tagged AKMT expressed under the control of the DHFR promoter, to create *flag-akmt complement-1* and *flag-akmt complement-2*.

### Loss of AKMT severely compromises the *T. gondii* lytic cycle

**Figure 3:**
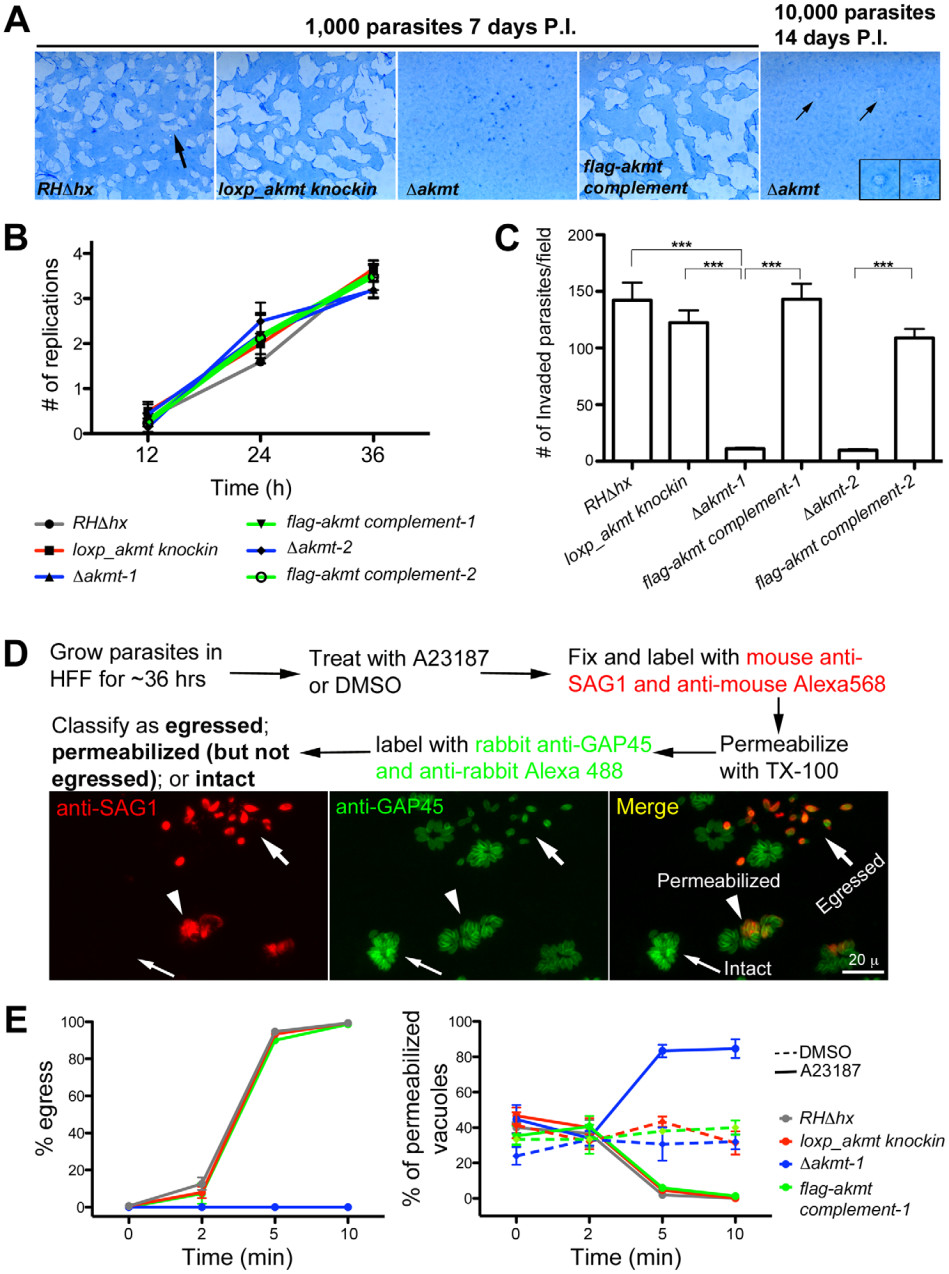
AKMT is important for invasion and egress, but not for parasite replication. (A) The lytic cycle of *Δakmt* parasites is greatly compromised. *Left*: Images showing portions of T12.5 tissue culture flasks containing a monolayer of HFF cells inoculated with 1000 parasites (*RHΔhx*, *loxp_akmt knockin*, *Δakmt* or *flag-akmt complement* parasites), grown for 7 days. The samples were then fixed and stained with Commassie Blue. The cultures were stained blue except for “plaques” (arrow) in which host cells had been destroyed by many rounds of parasite lytic cycles. No plaques were seen in the culture infected with *Δakmt* parasites. *Right*: HFF culture infected with 10,000 *Δakmt* parasites for 14 days. Only two small plaques (small arrows, insets) were detected. Insets 2X magnification. P.I.: Post Infection. (B) The loss of AKMT does not affect parasite replication. Parasites were grown for 12, 24 or 36 hrs and the number of parasites/vacuole was counted in 100 vacuoles/experiment. The average number of replications that had occurred at each time point was calculated from the results of three independent experiments. (C) The loss of AKMT results in a ∼90% decrease in invasion efficiency. The number of invaded parasites per field was counted in 10 fields each in 3 independent experiments. ***: P value <0.0001 (D) *Top*: Outline of A23187 induced egress assay. *Bottom*: Fluorescence images showing examples of three classes of parasitophorous vacuoles scored in the induced egress assay: 1) egressed if parasites had dispersed from the vacuole (large arrows); 2) permeabilized if parasites were retained in the parasitophorous vacuole but some or all of the parasites in the vacuole were labeled with anti-SAG1 antibody (arrowheads); 3) intact if none of the parasites in the vacuole were labeled with anti-SAG1 antibody (small arrows). (E) The loss of AKMT results in severe impairment in A23187 induced parasite egress despite efficient host cell permeabilization. *Left:* Percentage of vacuoles where egress had occurred 0, 2, 5, and 10 minutes after A23187 treatment. *Right:* Percentage of vacuoles that had been permeabilized 0, 2, 5, and 10 minutes after DMSO (dashed lines) or 5 µM A23187 (solid lines) treatment. Note that the AKMT expressing parasites (*RHΔhx*, *loxp_akmt knockin*, or *flag-akmt complement*) treated with A23817 have an apparent low percentage of permeabilized vacuoles at late time points (5 and 10 minutes), because most of the parasites have already egressed (see *left*). Total 50 vacuoles at each time point were scored for each of the three independent experiments. The behaviors of the two *Δakmt* lines were very similar, and both lines were fully complemented by FLAG-AKMT expression. For clarity, only the results from *Δakmt-1* and the corresponding complemented line, *flag-akmt complement-1* were included in the graph.

To evaluate how the lytic cycle of *Δakmt* parasites was affected by AKMT deficiency, confluent human foreskin fibroblast (HFF) monolayers were infected with the two parental strains (*RHΔhx* and *loxp_akmt knockin*), the *Δakmt* parasites, or the FLAG-AKMT complemented parasites, and the abilities of these parasites to form plaques were compared. Within seven days, 1000 parental or complemented parasites lysed more than 50% of the HFF monolayer (Figure 3A). In contrast, no plaques were observed when 1000 *Δakmt* parasites were used to infect the HFF monolayer for the same period of time, and an inoculum of 10,000 *Δakmt* parasites produced two small plaques in the monolayer after 14 days (Figure 3A), indicating that the lytic cycle of *Δakmt* parasites was severely compromised.

Because the lytic cycle of *T. gondii* is composed of host cell invasion, parasite replication and egress, we then assessed which step(s) of the lytic cycle is affected by AKMT depletion.

### Depletion of AKMT does not affect parasite replication

To assess intracellular replication, the two parental strains (*RHΔhx* and *loxp_akmt knockin*), the *Δakmt* parasites, and the FLAG-AKMT complemented parasites were grown for 12, 24 or 36 hours, and the number of intracellular parasites in each of 100 vacuoles was determined for each time point. We found no significant difference in intracellular replication rates among these parasite lines when the average number of replications that had occurred at 12, 24 and 36 hours post-infection were determined (Figure 3B). Therefore, AKMT depletion does not measurably perturb parasite replication.

### Depletion of AKMT causes highly impaired host cell invasion and parasite egress due to defective motility

Invasion is a multi-step process involving parasite movement along the host cell surface, attachment, and finally penetration to establish the parasitophorous vacuole. The invasiveness of the two parental strains (*RHΔhx* and *loxp_akmt knockin*), the *Δakmt* parasites, and the FLAG-AKMT complemented parasites was determined using the dual-color invasion assay developed previously [49], [50]. Specifically, 1×10^7^ parasites were added to confluent HFF monolayers and allowed to invade for 60 minutes. After fixation, extracellular parasites (*i.e.* parasites that had not yet penetrated the host cell) were first labeled with mouse anti-SAG1 antibody (which recognizes the major surface antigen on the outer surface of the plasma membrane of *T. gondii*), and a goat-anti-mouse Alexa 568 antibody. The samples were then permeabilized using TX-100, and all parasites were labeled with a rabbit anti-IMC1 antibody (a kind gift from Dr. Con Beckers at University of North Carolina, Chapel Hill) and then goat-anti-rabbit Alexa 488 antibody. The number of intracellular (*i.e.* invaded) parasites was then determined by counting parasites that were labeled for IMC1 but not labeled for SAG1. Using this assay, we found that AKMT depletion resulted in more than 90% decrease in invasion efficiency, indicating that AKMT plays a critical role in invasion (Figure 3C).

**Figure 4:**
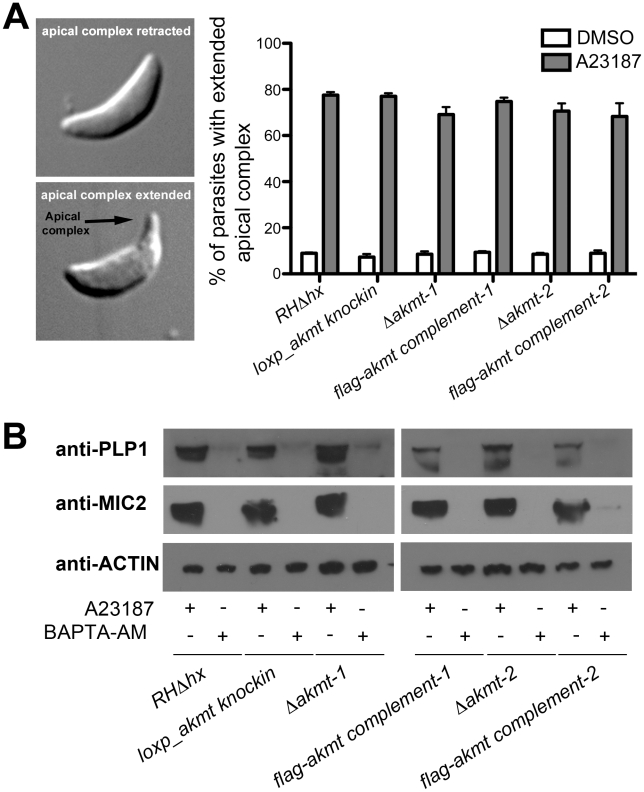
Ca^2+^ influx induced apical complex extension and microneme secretion are not affected in extracellular *Δakmt* parasites. (A) A23187 stimulated apical complex extension is not affected in extracellular *Δakmt* parasites. *Left*: DIC images of *T. gondii* with the cytoskeletal apical complex (arrow) retracted (top) or extended (bottom). *Right:* Percentage of parasites with extended apical complex when treated with DMSO (white bars) or 5 µM A23187 (gray bars). 300 parasites/slide were scored for apical complex extension in each of three independent experiments. (B) Microneme secretion assays analyzed by Western blot showing that the A23187 stimulated secretion of two microneme proteins, TgPLP1 (one of the proteins responsible for permeabilizing the host cell during parasite egress) [16] and TgMIC2 (one of the adhesive components of the motility apparatus) [11], [18], [24], [71], [72], is not affected in extracellular *Δakmt* parasites. Treatment with BAPTA-AM, a cell-permeant calcium chelator that inhibits microneme secretion [15], was used as the negative control for the secretion assay. Actin was used as the loading control.

To determine the role of AKMT in egress, parasites were first allowed to invade confluent HFF monolayers and replicate for ∼36 hours. We then induced egress with the calcium ionophore, A23187, which elevates parasite intracellular [Ca^2+^] (Figure 3D–E). For the parental and the FLAG-AKMT complemented parasites, after treatment with 5 µM A23187 for 5 minutes, parasites in ∼95% of vacuoles had dispersed (Figure 3E, left). In contrast, parasite dispersion occurred in less than 1% of the vacuoles containing *Δakmt* parasites after treatment with A23187 for 10 minutes (Figure 3E, left). Interestingly, we found that although *Δakmt* parasites failed to disperse from the host cell after 10 minutes of A23187 treatment, they efficiently permeabilized the infected host cells during this time frame (Figure 3E, right). Specifically, using the assay outlined in Figure 3D, we found that some or all of the *Δakmt* parasites in ∼90% of the vacuoles were positively labeled with anti-SAG1 antibody without detergent permeabilization. This percentage was significantly higher than those of the negative controls, *i.e.* DMSO (solvent for A23187) treated samples. This indicates that the dramatic delay in dispersion from the host cell was not caused by a generic block in Ca^2+^ signaling in the *Δakmt* parasites, as they can still respond to the Ca^2+^ influx by permeabilizing the host cell, most likely through protein secretion from micronemes. We also examined two calcium sensitive processes, the extension of the cytoskeletal apical complex and the secretion from micronemes, in extracellular parasites, and found that they were induced as efficiently in *Δakmt* as in the parental and the complemented parasites by A23187 treatment (Figure 4).

**Figure 5:**
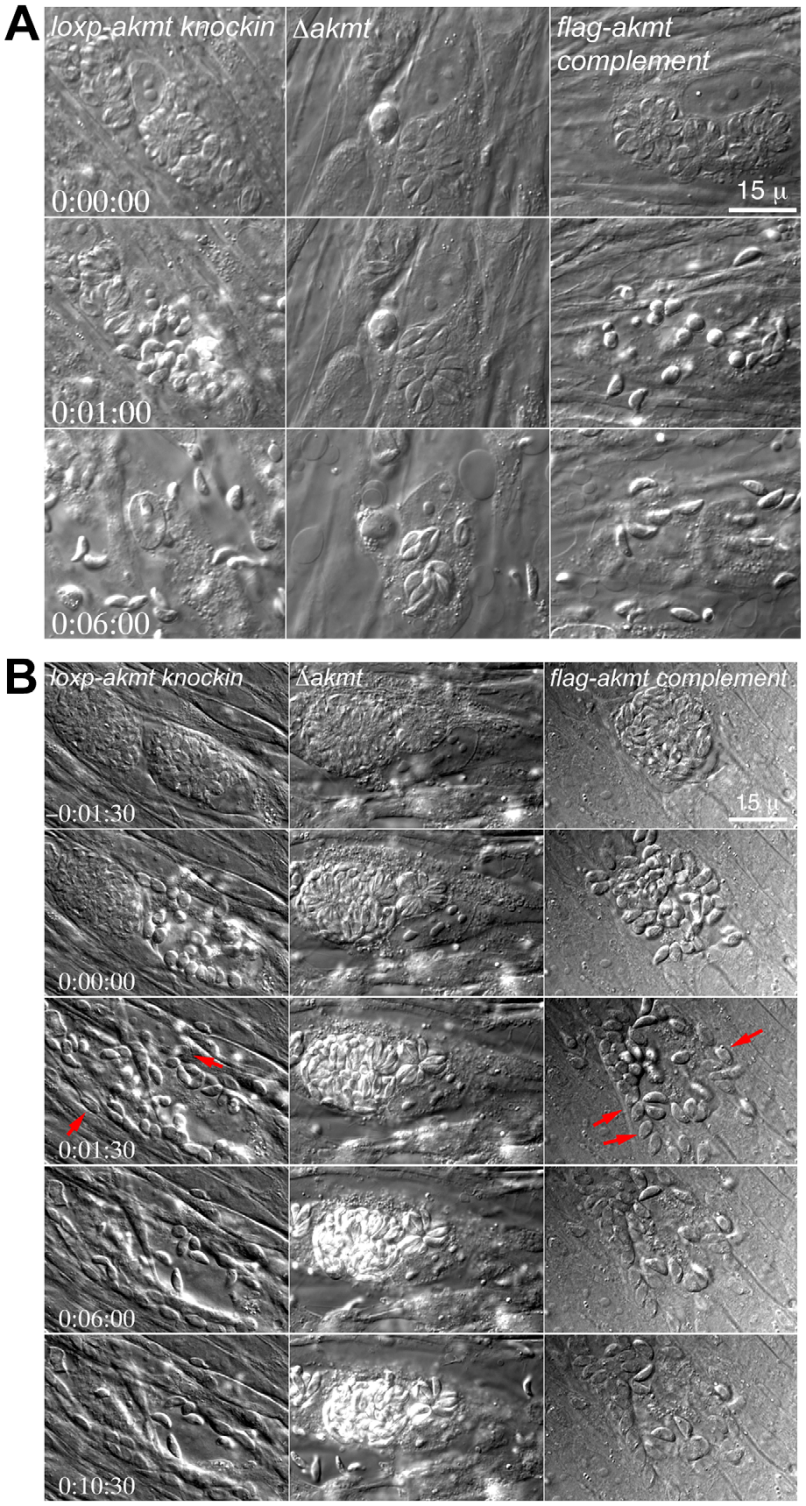
AKMT regulates parasite motility activation. (A) Images selected from time-lapse experiments of intracellular *loxp_akmt knockin*, *Δakmt* and *flag-akmt complement* parasites treated with 5 µM A23187 (also see Video S1). (B) Images selected from natural egress time-lapse experiments of intracellular *loxp_akmt knockin*, *Δakmt* and *flag-akmt complement* parasites (also see Video S2). To display the egress process in synchrony, the time point at which parasite egress (for *loxp_akmt knockin* and *flag-akmt complement* parasites) or host cell permeabilization (for *Δakmt* parasites) occurs is chosen as the 0∶00∶00 time point for all videos. Notice that for *loxp_akmt knockin* and *flag-akmt complement* parasites, host cell permeabilization and parasite egress occur almost simultaneously and many parasites (red arrows) invade adjacent host cells immediately after egress. *Δakmt* parasites however, remain largely immotile and fail to exit the host cell actively.

The failure of *Δakmt* parasites to disperse from the vacuole despite efficient host cell permeabilization suggested that activation of motility in these parasites was impaired. This hypothesis was confirmed by live video microscopy of A23187 induced-egress (Figure 5A and Video S1). For parental and complemented parasites, the increase in motility and the permeabilization of the parasitophorous vacuole occurred almost simultaneously. In contrast, even after the permeabilization of the host cell (indicated by the more refractile appearance of the parasites), *Δakmt* parasites remained largely non-motile and did not actively exit the host cell (Video S1).

**Figure 6:**
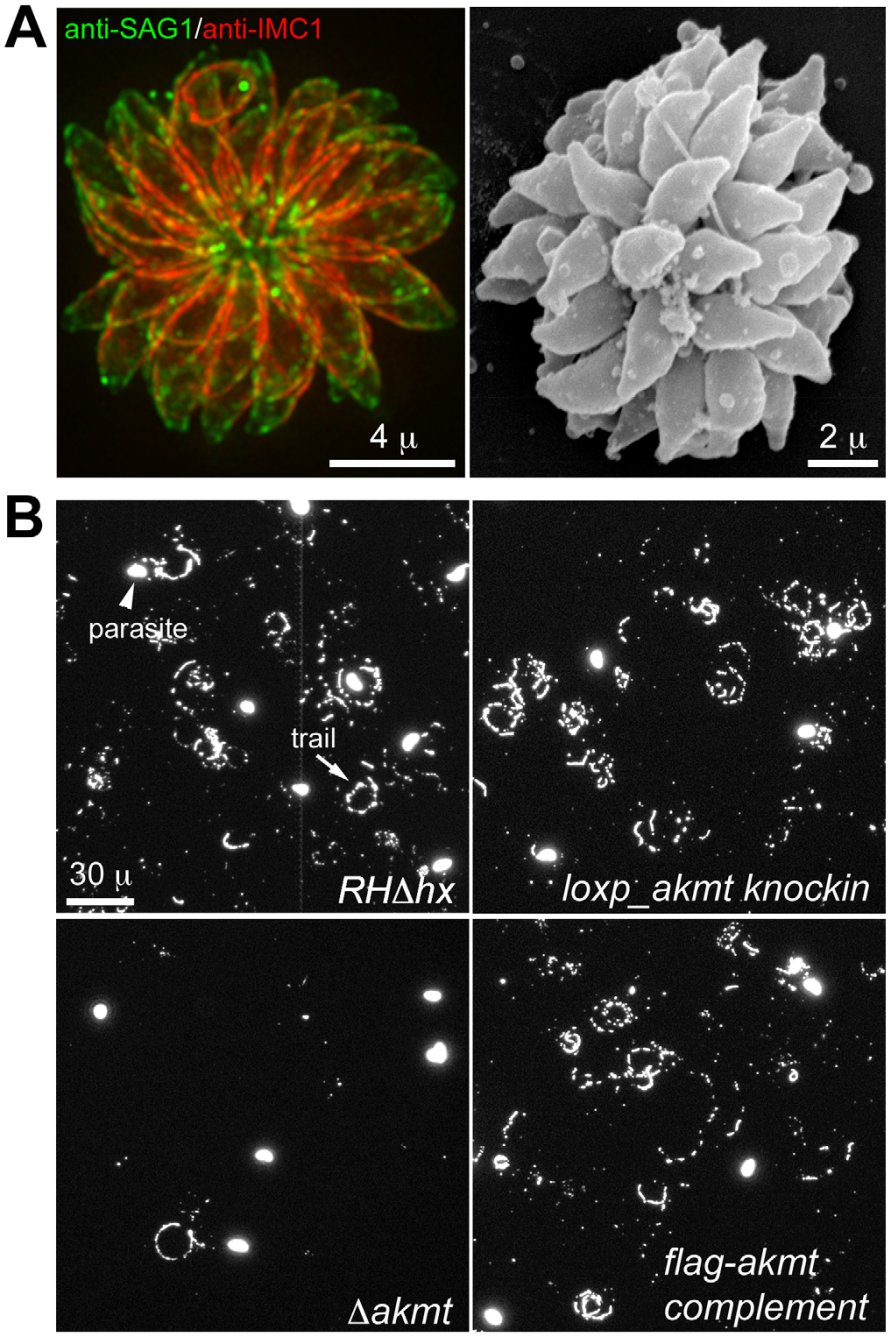
The motility of *Δakmt* parasites is impaired. (A) “Extracellular rosettes” found in *Δakmt* parasite culture. *Left*: Fluorescence image of a *Δakmt* extracellular rosette, a cluster of parasites remaining attached to each other after host cell breakdown. *Green*: anti-SAG1; *red*: anti-IMC1. *Right*: Scanning electron microscopy image of a *Δakmt* extracellular rosette. (B) Trail assays of *RHΔhx*, *loxp_akmt knockin*, *Δakmt* and *flag-akmt complement* parasites, showing that there is a marked decrease in trail deposition in *Δakmt* parasites compared to the parental and complemented lines. Mouse anti-SAG1 antibody and goat anti-mouse Alexa488 were used to visualize trails (arrow) deposited by the parasites.

Similar phenotype was observed in live video microscopy of natural (*i.e.* uninduced) egress (Figure 5B and Video S2; total 34, 15, and 24 vacuoles analyzed for *loxp_akmt knockin*, *Δakmt*, and *flag-akmt complement* parasites respectively). Interestingly, we found that groups of *Δakmt* parasites remained attached to each other after host cell breakdown, maintaining the “rosette” arrangement (Figure 6A) normally only seen when parasites are growing in the host cell (*c.f.* Figure 2B). This might be related to the impaired motility in these parasites. Alternatively, AKMT depletion might also cause defects in parasite detachment during egress.

We also confirmed the motility defects of *Δakmt* parasites using gliding motility assays. This assay takes advantage of the fact that *T. gondii* and other apicomplexan parasites leave protein trails behind as they glide along a solid substrate [17]. The trails formed by gliding *T. gondii* parasites contain the major surface antigen, SAG1, therefore can be visualized by immunofluorescence using anti-SAG1 antibody. The number of trails deposited is a qualitative indicator of parasite motility. We found that much fewer trails were deposited by the *Δakmt* parasites in comparison with the parental and complemented parasites when the same number of parasites were placed on serum coated coverslips and allowed to glide (Figure 6B), further confirming that the motility of *Δakmt* parasites is severely compromised.

### The lysine methyltransferase activity of AKMT is required for its function

**Figure 7:**
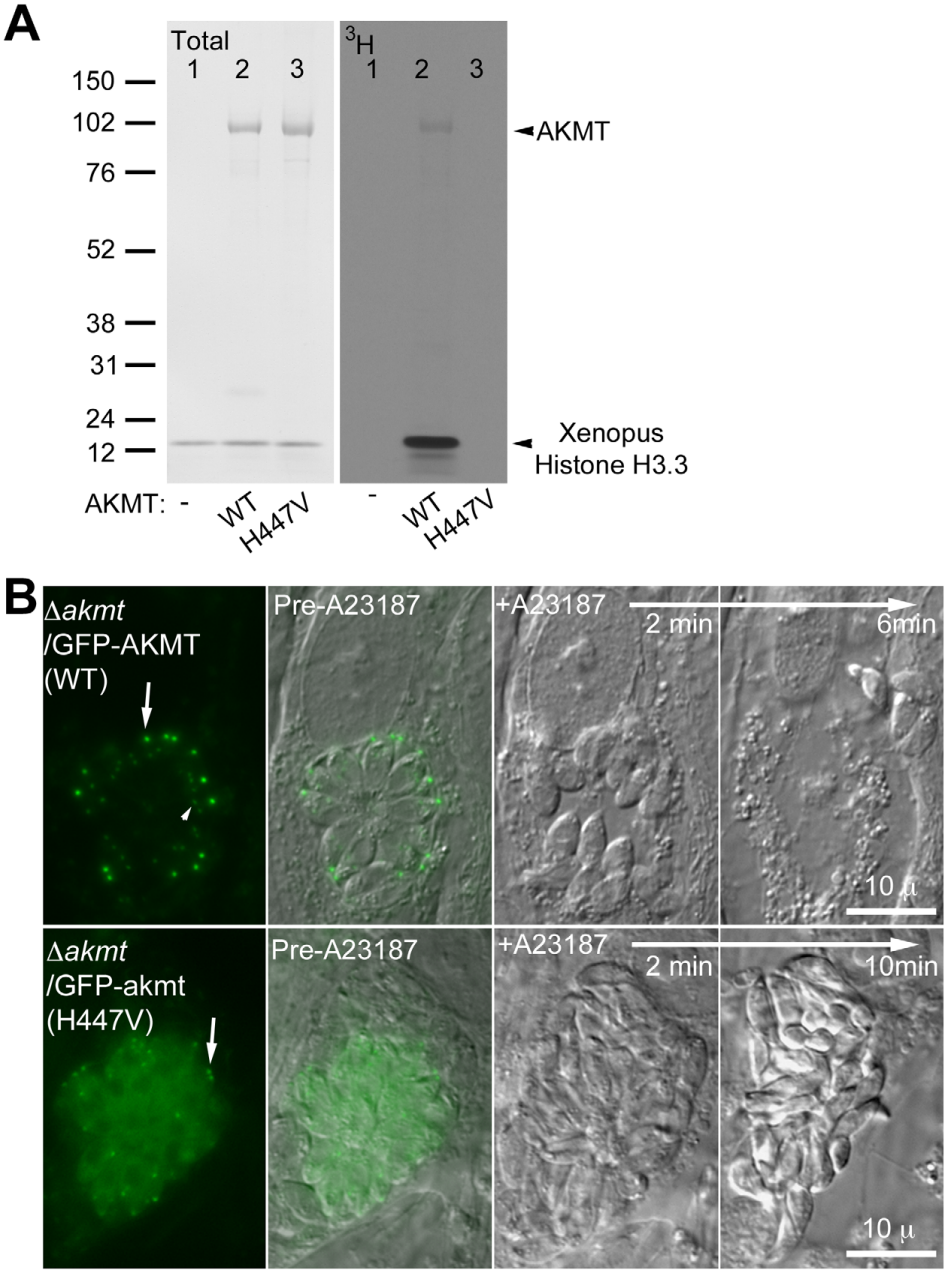
The lysine methyltransferase activity of AKMT is required for its function. (A) The mutation H447V abolished the enzyme activity of AKMT *in vitro*. *Left*: Blot stained with amido black to show total protein of histone H3.3+^3^H-SAM (lane 1); histone H3.3+1 µg FLAG-AKMT(WT)+^3^H-SAM (lane 2); and histone H3.3+1 µg FLAG-akmt(H447V)+^3^H-SAM (lane 3). *Right*: ^3^H signal of the same blot. Arrowheads indicate the positions of AKMT and histone H3 on the blots. Note that in the autoradiograph of FLAG-AKMT(WT) PKMT reaction (lane2), there is a weak band corresponding to AKMT self-methylation. (B) eGFP-akmt(H447V) failed to complement the egress defect of *Δakmt* parasites. Images show the results from an induced egress time-lapse experiment of intracellular *Δakmt* parasites expressing eGFP-AKMT(WT) (top) or eGFP-akmt(H447V) (bottom) parasites treated with 5 µM A23187. Arrows: apical complex of mature parasites. Arrowhead: daughter apical complex.

To determine if the PKMT enzymatic activity of AKMT is important for its function in activating motility, we created an enzymatically inactive AKMT by mutating a highly conserved histidine at amino acid 447 (H447) in motif III (predicted to be important for SAM binding, *c.f.* Figure 1B) to valine, a bulky, non-polar residue. This mutation completely abolished the methyltransferase activity of AKMT in the *in vitro* methylation assay (Figure 7A). eGFP tagged AKMT alleles were then transiently expressed under the control of the DHFR promoter in *Δakmt* parasites. Similar to endogenous AKMT (*c.f.* Figure 1), transiently expressed eGFP-AKMT(WT) (*i.e.* eGFP tagged wild-type allele of AKMT) was localized predominantly to the apical complex of the intracellular parasites (Figure 7B, top). EGFP-akmt(H447V), although present in the apical complex, had a more prominent cytoplasmic pool (Figure 7B, bottom), a pattern not observed in parasites transiently expressing eGFP-AKMT(WT). This suggests that the correct localization of AKMT may be dependent on its enzymatic activity, by a mechanism that is not yet understood.

To investigate if the lysine methyltransferase activity is required for AKMT function, intracellular *Δakmt* parasites transiently expressing eGFP-AKMT(WT) or eGFP-akmt(H447V) were treated with 5 µM A23187 and induced egress was observed for ∼20–30 minutes after the treatment. EGFP-AKMT(WT) expressing parasites in more than 95% of the vacuoles (71 out of 73 vacuoles examined) became highly motile and successfully dispersed from the host cell (Figure 7B, top). However all eGFP-akmt(H447V) expressing parasites (total 51 vacuoles examined) remained largely non-motile and failed to disperse (Figure 7B, bottom), similar to the phenotype previously observed with *Δakmt* parasites. The lysine methyltransferase activity of AKMT, therefore, is required for its function in promoting parasite motility. Also like *Δakmt* parasites, the parasites expressing eGFP-akmt(H447V) became refractile upon A23187 treatment (Figure 7B, bottom), suggesting that these parasites could secrete proteins that permeabilize the host cell despite impaired motility.

### Ca^2+^ signaling triggers the dispersal of AKMT from the apical complex before parasite egress

**Figure 8:**
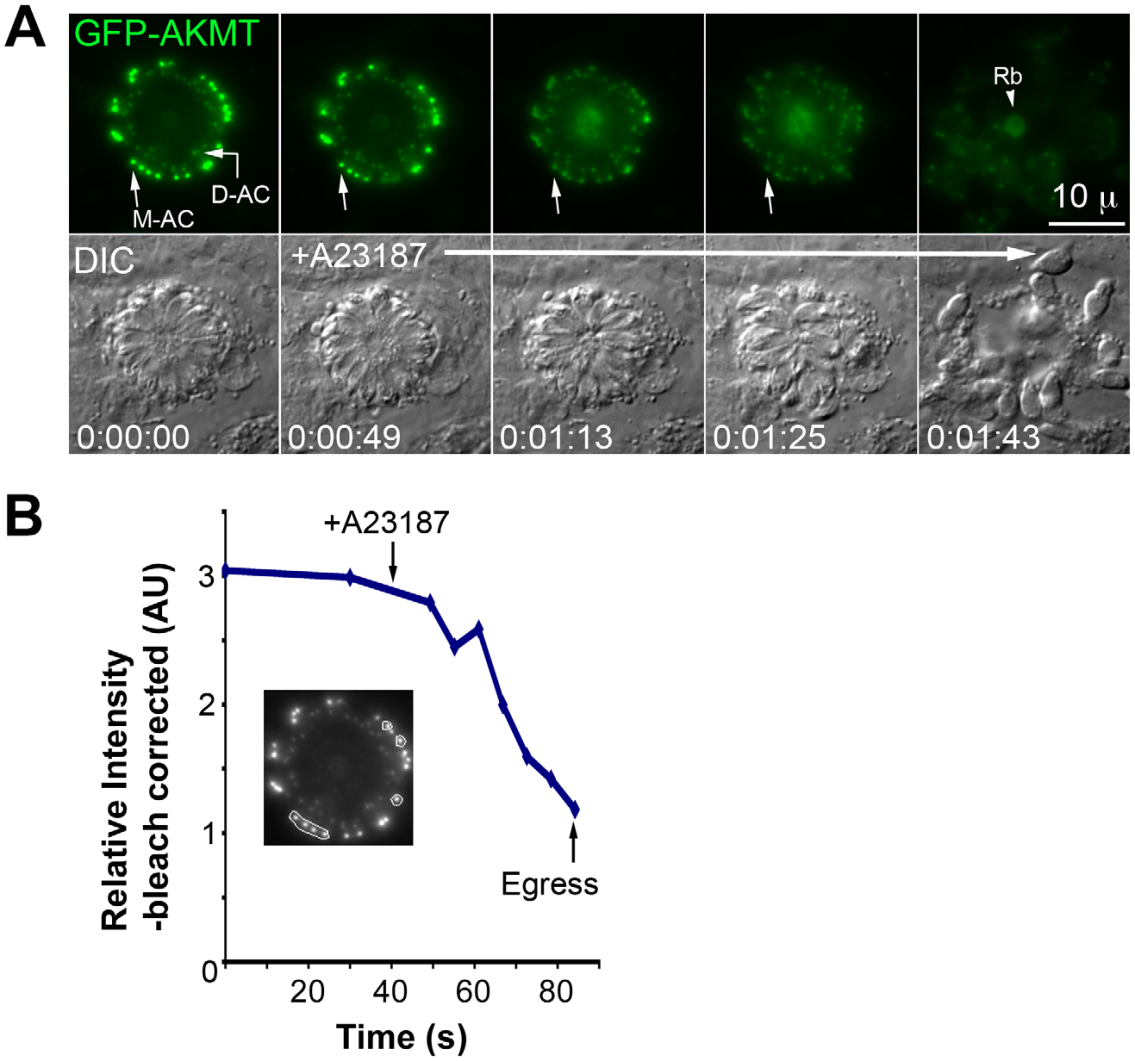
Elevated [Ca^2+^] triggers the dispersal of AKMT from the apical complex before A23187 induced parasite egress. (A) Images show the result from a time-lapse experiment of intracellular *Δakmt* parasites expressing eGFP-AKMT(WT) treated with 5 µM A23187 (A23187 was added between 0∶00∶30 and 0∶00∶49). These parasites were in the process of daughter construction as indicated by the two internal AKMT positive spots (daughter apical complexes: *D-AC*) in addition to the AKMT labeling in the mother apical complex (*M-AC*) of each parasite (*c.f.* Figure S1C). These parasites are connected through a structure in the central region called the residual body (*Rb, arrowhead*) [4]. Upon treatment with A23187, eGFP-AKMT relocated from the mother apical complex to the parasite body, followed by parasite egress. The residual body, containing some of the eGFP-AKMT released from the apical complex, is left behind. (B) Plot of average intensity (bleach-corrected) of eGFP-AKMT over time for seven mother apical complexes (encircled in the gray-scale image) in the experiment shown in A, showing the dissociation of AKMT from the mother apical complex before parasite egress. AU: Arbitrary Unit.

It has been shown that during natural parasite egress, the decrease in [K^+^] in host cell cytoplasm stimulates the increase in [Ca^2+^] in the parasite cytoplasm [8], a trigger that can be replaced by calcium ionophore treatment. Interestingly, we found that when the non-motile intracellular parasites were treated with 5 µM A23187, AKMT rapidly departed from the apical complex before parasite motility activation and egress (Figure 8 and Video S3).

**Figure 9:**
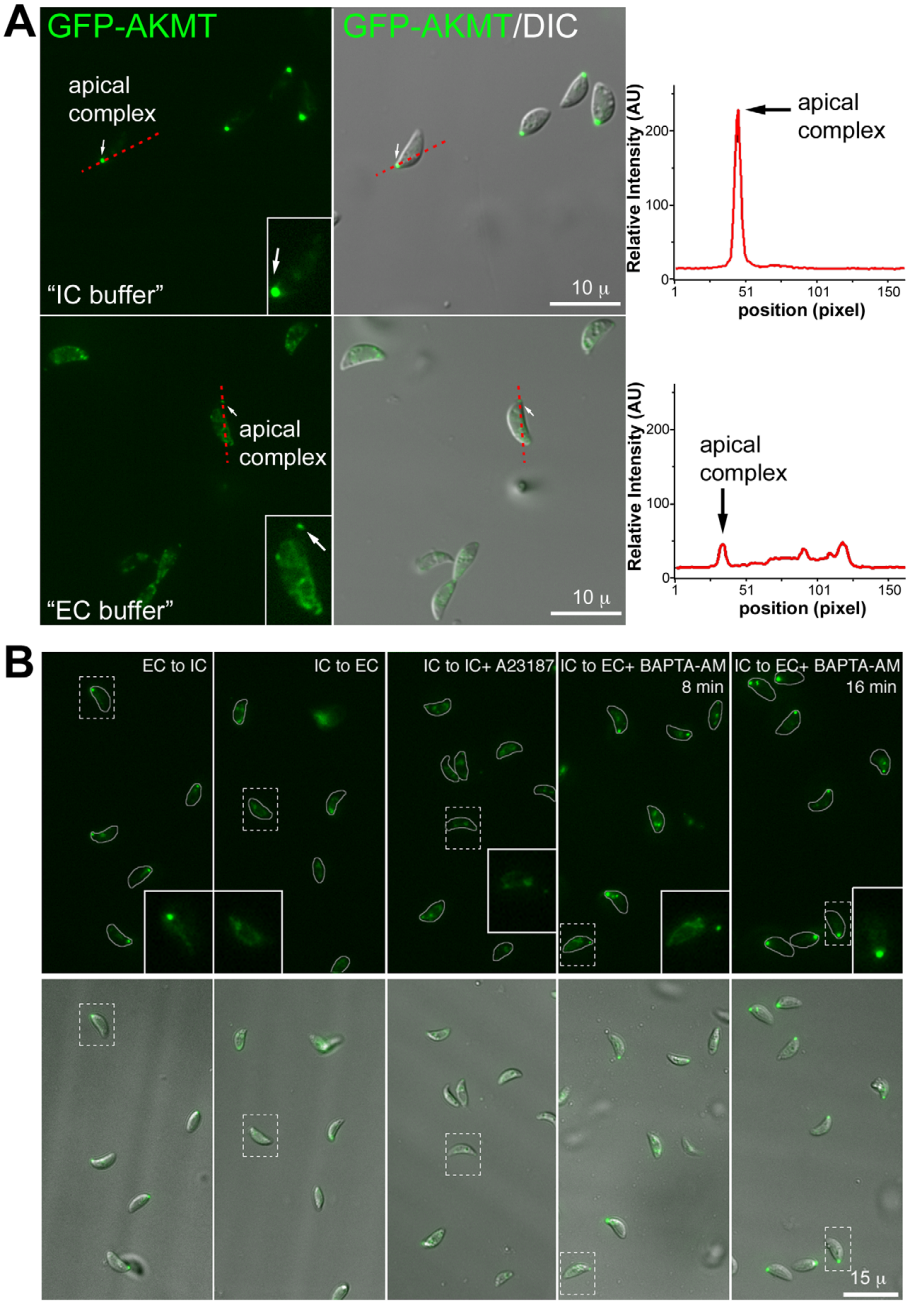
The localization of AKMT is affected by the extracellular and intracellular ionic composition. (A) *Left*: Images show the localization of AKMT in extracellular *egfp-akmt complement* parasites incubated either in a potassium-based buffer mimicking intracellular conditions (“IC buffer”, *top*) or a sodium-based buffer mimicking extracellular conditions (“EC buffer”, *bottom*). Inset 2X magnification. *Right*: Intensity plots along the red dashed lines in the images shown on the left. AU: Arbitrary Unit. (B) Images from buffer exchange experiments where extracellular *egfp-akmt complement* parasites were first incubated in EC or IC buffer for 25–30 minutes, and then were resuspended in buffer containing different ionic composition or drugs that affect intra-parasite [Ca^2+^] concentration. From left to right showing: “EC to IC”- image of *egfp-akmt complement* parasites ∼32 minutes after buffer exchange from EC to IC buffer, which resulted in redistribution of AKMT from parasite body to the apical complex, a process that took ∼30 minutes to complete; “IC to EC”- image of *egfp-akmt complement* parasites ∼7 minutes after buffer exchange from IC to EC buffer, showing eGFP-AKMT dissociated from the apical complex; “IC to IC+A23187”- image of *egfp-akmt complement* parasites ∼5 minutes after buffer exchange from IC to IC buffer +5 µM A23187, showing eGFP-AKMT dissociated from the apical complex; “IC to EC+BAPTA-AM”- images of *egfp-akmt complement* parasites 8 and 16 minutes after buffer exchange from IC buffer to EC buffer+50 µM BAPTA-AM, showing a gradual relocation of AKMT from the parasite body to the apical complex. This change was noticeable by 7–8 minutes, and completed by ∼15–16 minutes after the buffer exchange. Top: fluorescence images super-imposed with the outlines of the parasites (gray borders). Bottom: overlay images of fluorescence and DIC. Inset 2X magnification

We then investigated if the two distinct patterns of AKMT distribution, apparently sensitive to intra-parasite [Ca^2+^], could be interconnected by manipulating extra-parasite ionic environment. Extracellular parasites stably expressing eGFP-AKMT in a *Δakmt* background (*egfp-akmt complement*) were incubated in either a potassium-based buffer mimicking intracellular conditions (“IC buffer”, [8], [51]) or a sodium-based buffer mimicking extracellular conditions (“EC buffer”, [8]) (Figure 9A). We found that eGFP-AKMT was concentrated in the apical complex when the parasites were incubated in IC buffer (Figure 9A, top), whereas most eGFP-AKMT was present in the parasite body when the parasites were incubated in EC buffer (Figure 9A, bottom). To further investigate the dependency of eGFP-AKMT localization on external and internal ionic compositions, we carried out a series of buffer exchange experiments (Figure 9B). Extracellular parasites were first incubated in EC or IC buffer for 25–30 minutes, and then resuspended in buffer containing different ionic composition or drugs that affect intra-parasite [Ca^2+^] concentration. Exchange from EC to IC buffer resulted in redistribution of AKMT from parasite body back to the apical complex, a process that took ∼30 minutes to complete (an image from the 32 minute time-point is shown in Figure 9B). Interestingly, it took significantly less time for the reverse process to occur. When IC buffer was exchanged with EC buffer, eGFP-AKMT dissociated from the apical complex within 3 minutes, and this pattern persisted through the remaining ∼25 minutes of the experiment (an image from the 7 minute time-point is shown in Figure 9B). This dispersion could also be triggered by calcium ionophore treatment, when IC buffer was replaced with IC buffer containing 5 µM A23187 (an image from the 5 minute time-point is shown in Figure 9B). Interestingly, when IC buffer was replaced with EC buffer containing 50 µM BAPTA-AM (a cell-permeant Ca^2+^ chelator), although most eGFP-AKMT initially dispersed to the parasite body (data not shown), its localization became more and more concentrated in the apical complex over time. This change was noticeable by 7–8 minutes (Figure 9B); and by ∼15–16 minutes after the buffer exchange, eGFP-AKMT was again predominantly located in the apical complex (Figure 9B). Taken together, our data suggest that localization of AKMT is sensitive to the changes in ionic composition in the parasite environment as well as intra-parasite [Ca^2+^].

**Figure 10:**
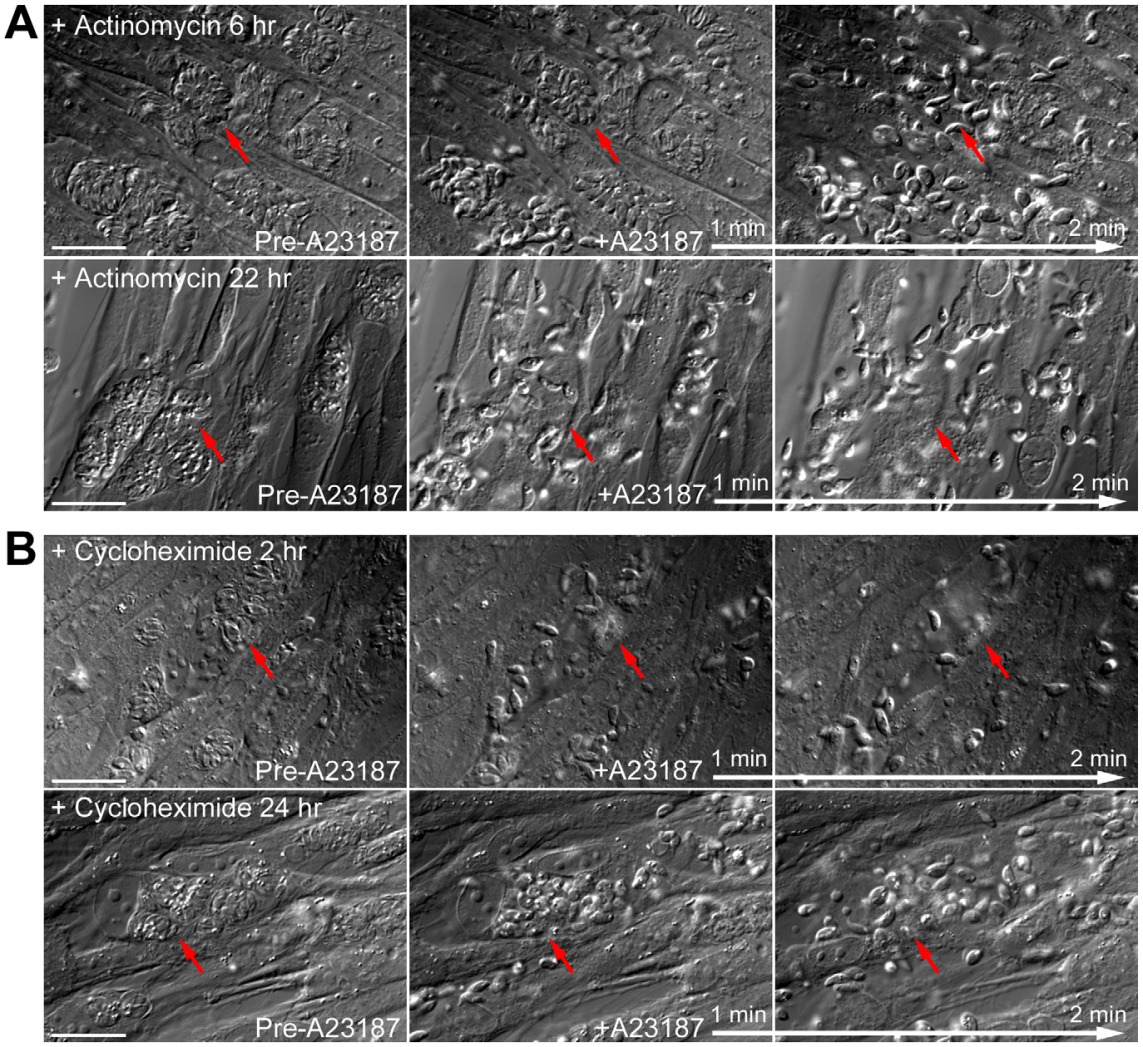
New RNA transcription and protein synthesis is not required for motility activation in induced egress. (A) Images show the results from time-lapse experiments of *RHΔhx* parasites treated with 0.5 µg/ml actinomycin D (an inhibitor of RNA transcription) for 6 (top) or 22 hours (bottom) showing that this treatment has no effect on parasite motility stimulated by 5 µM A23817. Red arrows provide reference points for following changes of the parasitophorous vacuoles indicated in each sequence. Note the obvious lack of parasite growth in the presence of actinomycin D, comparing the size of the parasitophorous vacuoles in the culture treated for 6 versus 22 hours (Actinomycin D treatment for the two cultures was initiated simultaneously ∼26 hours post-infection.). Bars = 20 µm. (B) Images show the results from time-lapse experiments of *RHΔhx* parasites treated with 10 µM cycloheximide (an inhibitor of protein translation) for 2 (top) or 24 hours (bottom) showing that this treatment has no effect on parasite motility stimulated by 5 µM A23817. Red arrows provide reference points for following changes of the parasitophorous vacuoles indicated in each sequence. Bars = 20 µm.

Methylation of specific lysine residues on nuclear proteins, including histones and transcription factors such as P53, is a post-translational modification that has a profound impact on the regulation of gene transcription [35]–[40], [52]–[55]. It has been reported previously that the motility of mammalian cells can be regulated by the status of histone lysine methylation through transcriptional control of the proteins involved in motility, such as integrins [56], [57]. The relocation of AKMT upon Ca^2+^ influx raises the possibility that a small number of AKMT molecules might enter the nucleus after release from the apical complex and affect gene expression to control parasite motility. If this is true, then blocking RNA transcription or protein synthesis should inhibit the Ca^2+^-stimulated parasite motility. We therefore examined the effect of actinomycin D treatment in A23187 induced egress. Measurement of uracil [5,6-^3^H] incorporation into total RNA showed that ∼5.5 hr treatment of 0.5 µg/ml actinomycin D inhibited RNA transcription by ∼98% to 100%. However, after incubation in 0.5 µg/ml actinomycin D for ∼6, 8, 22 or 30 hours (# of vacuoles examined-173, 13, 86 and 33, respectively), *RHΔhx* parasites in >99% (*i.e*. 304 out of 305) of the vacuoles actively dispersed from the host cell upon stimulation by 5 µM A23187 within the 5 to 10 minute period of observation (Figure 10A). Similarly, 10 µM cycloheximide treatment, which inhibits protein synthesis by ∼98% within 2 hours [25], [58], had no detectable effect on parasite motility activation. After treatment with 10 µM cycloheximide for 2 or 23–24 hours (49 and 67 vacuoles examined, respectively), *RHΔhx* parasites in >99% (*i.e*. 115 out of 116) of the vacuoles actively dispersed upon exposure to 5 µM A23187 (Figure 10B). Taken together, our data suggests that new gene expression is not required for parasite motility activation and thus AKMT is unlikely to regulate parasite motility through controlling gene expression.

## Discussion

During egress, non-motile parasites become motile in response to an increase in their cytoplasmic [Ca^2+^] [2], [8], [10]–[15]. This transition is necessary for the parasite to rapidly exit the dying host cell and invade into adjacent hosts. It has been proposed that this trait is highly advantageous for the parasite in an *in vivo* setting, helping it to evade attack from the immune system [13]. The activation of *T. gondii* motility therefore is vital for parasite survival and disease pathogenesis. Although the force-generating apparatus underlying motility in *T. gondii* has been extensively studied [14], [17]–[27], the signaling cascade controlling this process is not well characterized. In this work, we identified a new protein, AKMT, which is important for regulating the transition from immotile to motile in the parasite lytic cycle. We found that AKMT is an active lysine methyltransferase enzyme and that this enzymatic activity is required for its function in activating parasite motility. Interestingly, AKMT is localized to the apical complex in intracellular, non-motile parasites, but is released and disperses throughout the parasite immediately prior to the onset of motility and egress.

In order to study the function of AKMT in *T. gondii*, an AKMT deletion mutant (*Δakmt*) was generated using a Cre-LoxP approach [46], [47] previously implemented in our laboratory [48]. This strategy consists of two steps. The first step selects for homologous recombinants after transfecting with a construct containing the AKMT 5′UTR, two LoxP sites flanking the AKMT coding region plus a HXGPRT expression cassette, and the AKMT 3′UTR. The second step selects for the knock-out mutant after transient expression of the Cre recombinase in the homologous recombinant. Importantly, the loss of the target gene coding sequence via recombination by Cre recombinase simultaneously results in the loss of HXGPRT, which allows for the enrichment of the knockout mutants through a subsequent 6-TX selection that drastically slows the growth of parasites where recombination has not occurred. This method was used previously to characterize the function of TgMORN1 [48], a protein important for parasite cytokinesis [48], [59]. Our success in generating *Δakmt* parasites, which has severe defects in both invasion and egress due to motility impairment, demonstrates that this Cre-LoxP method is versatile and can be used for studying the function of genes that are important for different aspects of *T. gondii* lytic cycle.

How does AKMT regulate parasite motility? We note that the phenotype caused by loss of AKMT, *i.e.* defective invasion and egress due to impaired motility, but normal intracellular replication, is very similar to those reported for parasites deficient in MyoA or GAP45, core components of the motility apparatus [20], [27]. It is thus conceivable that through protein lysine methylation, AKMT regulates, directly or indirectly, the functional state of the motility apparatus to control the switch from immotile to active gliding motility.

Methylation of specific lysine residues was first described and well characterized as a modification of histones, which controls gene transcription [35]–[42]. In recent years, more and more examples of lysine methylation in non-histone proteins have been discovered [60], [61]. AKMT can methylate lysine residues on an artificial substrate, Xenopus histone H3.3, *in vitro*, however, we think that it is unlikely to regulate parasite motility by controlling gene expression through histone methylation for the following reasons. First, in intracellular, non-motile parasites, AKMT is specifically localized to the apical complex, but undetectable in the nucleus. In stark contrast, two canonical histone lysine methyltransferases characterized in *T. gondii*, TgSET8 and TgKMTox, are both chromatin binding proteins highly enriched in the nucleus [41], [42]. Second, before parasite egress, AKMT is released from the apical complex to the parasite body, thus some AKMT molecules might go into the nucleus after this release and change gene expression. However, we found that inhibition of new RNA transcription or protein synthesis has no effect on motility activation during egress. This argues that new gene expression is not required for activating parasite motility, and thus AKMT does not regulate motility through modifying gene expression. Therefore we favor an alternative model and propose that protein lysine methylation by AKMT has a direct effect on parasite motility.

Specifically, we speculate that two unusual characteristics of AKMT are important for its regulation of motility – its unconventional enzymatic domain and its dynamic relocalization. The SET domain in canonical PKMTs contains four highly conserved motifs: motif I for SAM binding; motif II for catalysis of methyl transfer; and motif III and IV for SAM binding and formation of target lysine-binding channel [33], [34]. AKMT, however, contains a novel SET domain, in which a conserved motif II cannot be found. This may be important for regulating AKMT activity and/or substrate specificity during the motility activation. In addition to the unusual enzymatic domain, we found that AKMT relocates from the apical complex to the parasite body before egress, suggesting that AKMT regulation of parasite motility might be accomplished by the precise temporal control of its localization in response to environmental changes. Our current working model is that AKMT is sequestered in the apical complex during the non-motile, intracellular state, which prevents this enzyme from interacting with its substrates in the cytoplasm (“cytoplasm” in this context includes the space containing the motility apparatus between the IMC and plasma membrane). Upon the increase in intra-parasite [Ca^2+^] (triggered by the drop of [K^+^] in its host cell), AKMT is released to the cytoplasm to interact with and methylate its substrate, which eventually activates parasite motility.

It is almost certain that this working model is oversimplified. For example, it is possible that the true effector of AKMT is present in the apical complex, which controls motility in a fashion that we cannot envision based on our current knowledge of the parasite motility regulation. Our simple model also does not account for the inefficient recruitment of the enzymatically dead AKMT to the apical complex in intracellular parasites, which suggests that lysine methyltransferase activity, or at least an intact enzyme active site, is necessary for proper localization of AKMT. However, whatever the mode of action, protein methylation by AKMT has to somehow affect the functionality of the motility apparatus, directly or indirectly. This is clearly shown by our result that the motility activation defect of the AKMT knockout mutant can be rescued by the expression of the wild-type, but not the enzymatically inactive AKMT allele. We speculate that the protein lysine methylation by AKMT may be critical for efficiently “engaging” the motility apparatus in the sense of a motor “engaging” a drive shaft via a clutch or other conditional mechanical coupling. Without the lysine modification, the engagement could still take place but is “sloppy”, thus occurs in a sporadic fashion and at a much lower frequency. Consequently, when AKMT is depleted, the efficiency of motility activation is significantly reduced, which results in defective invasion and egress. Interestingly, Ca^2+^ induced microneme secretion and host cell permeabilization appears to be normal in AKMT-null parasites. Thus AKMT functions in a previously unknown pathway that is downstream from or parallel to those mediated by TgCDPK1 and TgPKG, which control microneme secretion [28]–[31]. Therefore despite their highly compromised motility, AKMT-null parasites kill host cells through protein secretion and membrane permeabilization, and are passively released after the dead hosts disintegrate.

It is remarkable that the AKMT relocates from the apical complex to the parasite body in response to the increase in intra-parasite [Ca^2+^]. It has previously been shown that glycolytic enzymes important for energy generation during motility are translocated to the parasite periphery when the parasite transitions from the intracellular to the extracellular environment [25]. Therefore it seems to be an emerging theme that motility activation correlates with the temporal control of the localization of relevant proteins. We also notice that when intracellular parasites within the same vacuole are connected at their posterior ends through the residual body, the residual body can have a higher concentration of AKMT than the parasite body after AKMT release from the apical complex (*c.f.* Figure 8). As simple diffusion alone cannot build up a concentration gradient, this suggests that AKMT might move through the cytoplasm from the anterior to the posterior end of the parasite via a more complicated mechanism. We do not know the cause for this concentration difference, but we think that active transport is unlikely, as there is no known actin or microtubule based transport system that spans the entire length of the parasite. As the residual body often contains discarded and/or degraded parasite body parts leftover from previous cell divisions [4], [62], it is conceivable that it contains a higher concentration of AKMT binding sites, and thus retains the “over-flow” of AKMT after the binding sites in the parasite body are saturated.

AKMT orthologs, sharing the novel enzymatic domain of TgAKMT, are found in other apicomplexan parasites (*c.f.* Figure S1A), including *Plasmodium falciparum*, suggesting that a common pathway for AKMT based motility regulation may exist in these parasites, and therefore could be a target for treatment and preventative measures. In the future, it will be interesting to uncover the upstream signaling pathways that regulate the release of AKMT from the apical complex preceding parasite egress, to determine if this release is co-regulated with the localization changes of the glycolytic enzymes, to identify the substrates and other downstream effectors of AKMT, as well as to elucidate the function of AKMT orthologs in other important apicomplexan parasites.

## Materials and Methods

### Parasite culture and transfection

*T. gondii* tachyzoites derived from RH strain were used in all experiments. The parasites were maintained by continuous passage in human foreskin fibroblasts (HFFs) as previously described [63], [64]. AKMT knockout parasites, which display invasion and egress defects were cultured by “over-inoculation”. For maintaining lab culture, typically an inoculum of 1.5−2×10^7^ AKMT knockout parasites will lyse a monolayer of HFF host cell in a T12.5 flask after ∼3 days, yielding ∼5×10^7^ parasites. For comparison, a T12.5 flask infected with ∼1/5−1/10 as many wild-type parasites yields a similar final number of parasites ∼2 days after the infection.

For each *T. gondii* transfection, 25–50 µg of DNA was electroporated into 1−2×10^7^ extracellular parasites suspended in a potassium-based buffer (“intracellular (IC) buffer”) (120 mM KCl; 0.15 mM CaCl2; 10 mM KH2PO4/K2HPO4 pH 7.6; 25 mM HEPES-KOH pH 7.6; 2 mM K_2_EGTA pH 7.6; 5 mM MgCl2, pH adjusted with KOH) [51] containing 2 mM K_2_ATP and 5 mM glutathione. Electroporation was performed using a Harvard Apparatus BTX-BCM630 electroporator (Holliston, MA) with the electroporation voltage set at 1480 V, capacitance set at 50 µF, and resistance set at 25Ω.

### Construction of AKMT expression plasmids

#### *T. gondii* expression plasmids

pmin-eGFP-AKMT was created by amplifying the AKMT coding region from a RH cDNA library (obtained as previously described [32]) using S112 and A113 primers (Table S1) digested with BglII and AflII, and subcloned into pmin-eGFP-TgDLC plasmid [32] replacing the TgDLC/BglII-AflII fragment.

To create pTKO2_II_AKMT_CKO (LoxP_AKMT knockin plasmid), a ∼2 kb fragment downstream of the AKMT stop codon was first amplified using primers AKMT-3′UTR-S1 and AKMT-3′UTR-A1 (TableS1), digested with ApaI and NheI and ligated into pTKO2_II plasmid [48] (derived from PTKO, a kind gift from Drs Gusti Zeiner, Michael Reese and John Boothroyd at Stanford University), digested with NheI and ApaI, which resulted in the plasmid PTKO2_II_AKMT_3′UTR. Secondly, a ∼2 kb fragment 5′ of the AKMT coding region in the genome was amplified using primers AKMT-5′UTR-S1 and AKMT-5′UTR-A1 (Table S1) digested with EcoRI and NotI, and ligated into EcoRI and NotI sites of pTKO2_II_AKMT_3′UTR, which resulted in the plasmid pTKO2_II_AKMT_5′+3′UTR. Lastly, the AKMT coding sequence was amplified from pmin-eGFP-AKMT using primers AKMT-CDS-S1 and AKMT-CDS-A1 (13 bp of AKMT Kozak sequence was included in the primer AKMT-CDS-S1) (Table S1) digested with PmeI and RsrII and ligated into PmeI and RsrII sites of PTKO2_II_AKMT_5′+3′UTR to give the pTKO2_II_AKMT_CKO plasmid. Genomic DNA for amplifying the 5′ and 3′ UTR of AKMT was harvested from RH parasites using the Promega Wizard Kit (CAT#A1120: Promega).

pmin-FLAG-AKMT was created by excising the AKMT coding region from the pmin-eGFP-AKMT plasmid by BglII-AflII digestion and subcloned into pmin-FLAG-tubulinA1 (a kind gift from Dr. John Murray) replacing the tubulinA1/BglII-AflII fragment.

pmin-eGFP-AKMT301-709aa was created by amplifying the truncated AKMT coding sequence using primers AKMT-301F and A113 (Table S1) and digested with BglII and AflII and subcloned into pmin-eGFP-AKMT replacing the AKMT/BglII-AflII fragment.

To create pmin-eGFP-akmt(H447V), a 1352 DNA fragment (flanked with BglII and PstI) including nt4-1349 of AKMT containing the desired mutation was first synthesized and cloned into the PUC57simple plasmid (akmt(H447V)-N _PUC57simple, constructed by Genescript, Inc, New Jersey, U.S.A). The fragment was then isolated via BglII-PstI digestion and then subcloned into the BglII-PstI sites of pmin-eGFP-AKMT301-709aa to create pmin-eGFP-akmt(H447V).

#### Bacterial expression plasmids

To create pQE30-His-AKMT, a DNA fragment containing the AKMT coding sequence was first isolated from pmin-eGFP-AKMT by AflII digestion, the 5′ overhang filled in by Klenow-DNA polymerase fragment, followed by BglII digestion. To give pQE30-His-AKMT, this piece of DNA was then ligated into pQE30-DIP13 (a kind gift from Dr. Wolfgang Mages, Universität Regensburg, Germany, [65]), which was first digested with HindIII, blunt-ended through a fill-in reaction, and subsequently digested with BamHI.

To create pET22b-FLAG-AKMT, the AKMT coding sequence was amplified from pmin-eGFP-AKMT using primers NheI-AKMT-S and EcoRI-AKMT-A (Table S1) and the PCR product was digested with NheI and EcoRI. The PCR fragment was subcloned into the NheI and EcoRI sites of pET22b-FLAG-PolB vector (A kind gift from Tom Sladewski and Dr. Pat Foster, Indiana University; [66])where the PolB coding sequence was replaced with that of AKMT.

pET22b-FLAG-akmt(H447V) was created by a triple ligation of: 1) a PCR product using NheI-AKMT-S and M13 reverse (Table S1) as primers and akmt(H447V)-N _PUC57simple as template, digested with NheI and PstI; 2) a PCR product using AKMT-301F and EcoRI-AKMT-A (Table S1) as primers and pmin-eGFP-AKMT as template, digested with PstI and EcoRI; and 3) vector pET22b-FLAG-PolB digested with NheI and EcoRI.

### Generation of *loxp_akmt knockin*, Δ*akmt*, and complemented parasites

To create the *loxp_akmt knockin* parasites, 30 µg pTKO2_II_AKMT_CKO plasmid was first linearized by NotI digestion and transfected into ∼1×10^7^ *RHΔhx* parasites [64], and then selected with myocophenolic acid (MPA) (25 µg/ml) and xanthine (50 µg/ml) until approximately 75% of the parasites were GFP positive. GFP negative parasites were then collected by flow cytometry and three parasites were sorted into each well of a 96 well plate containing confluent HFF monolayers. Genomic DNA was extracted from single clones using Extract-N-Amp kit (CAT#XNAB-1kt: Sigma) and screened by genomic PCR for clones of parasites where the endogenous AKMT locus had been replaced by LoxP-AKMT-HXGPRT expression cassette-LoxP.

To create the *Δakmt* parasites, *loxp_akmt knockin* parasites were transfected with 25 µg pmin-Cre-eGFP plasmid to excise the fragment between the LoxP sites, and placed under 80 µg/ml 6-thioxanthine (6-TX) selection. After the third and fourth passage of the 6-TX resistant population, parasites were cloned by limiting dilution, and *Δakmt* parasites were first selected by immunofluorescence using a rat anti-AKMT antibody (see below) and subsequently confirmed by genomic PCR and Western blotting.

To generate the complemented lines, FLAG-AKMT complemented parasites (*i.e. flag-akmt complement*), 1×10^7^ *Δakmt* parasites were transfected with 25 µg of pmin-FLAG-AKMT plasmid and grown without applying any drug selection. After 21 days, 100% of the parasites were expressing FLAG-AKMT, which were then used for assessing the effectiveness of the complementation. eGFP-AKMT complemented parasites (*i.e. egfp-akmt complement*) were generated in the same way as FLAG-AKMT complemented parasites. We made several attempts to generate stable lines by this same method using the enzymatically dead mutant-akmt(H447V) instead of the enzymatically active form, but failed in all cases. pmin-FLAG-AKMT and pmin-eGFP-AKMT plasmids contain no *T. gondii* selectable marker. Therefore like *Δakmt* parasites, FLAG-AKMT and eGFP-AKMT complemented parasites are also HXGPRT deficient.

### Protein expression and purification

#### Expression and purification of His-AKMT

His-AKMT was purified from BL21 *E. coli* expressing pQE30-His-AKMT as previously described [48]. For generating the AKMT antibody, His-AKMT bound to Talon-resin (Clontech) was eluted with lysis buffer (8 mM Tris-Acetate pH 7.5, 3 mM Tris base pH unadjusted, 100 mM KAcetate, 1 mM MgAcetate) containing 500 mM imidazole.

#### Expression and purification of FLAG-AKMT

FLAG-AKMT was purified from BL21 *E. coli* expressing pET22b-FLAG-AKMT as described above for pQE30-His-AKMT with the following modifications. Bacteria were induced with 1 mM IPTG for ∼20 hours at 16°C and lysed in lysis buffer (8 mM Tris-Acetate pH 7.5, 7 mM Tris base pH unadjusted, 100 mM KAcetate, 1 mM MgAcetate) containing 0.5% Triton-100 (TX-100), protease inhibitor cocktail (CAT# P8465: Sigma) and 4 mM DTT. FLAG-AKMT was purified using anti-FLAG M2 agarose (CAT# A2220: Sigma) and was eluted from beads with 100 µg/ml FLAG-peptide (CAT# 3290: Sigma) in lysis buffer containing 4 mM DTT. Eluted proteins were dialyzed into dialysis buffer (lysis buffer containing 50% glycerol, 4 mM DTT) at 4°C overnight, aliquoted and stored at −20°C.

### Antibody production and affinity purification

Polyclonal rat anti-AKMT was produced by Cocalico Biological, Inc using His-AKMT recombinant protein (generated as described above) as antigen. Affinity purification of AKMT antibody was performed as previously described [48], [67].

### Western blot

Western blots were performed as previously described [48]. All antibody incubations were carried out in TBS (20 mM Tris base pH 7.4, 150 mM NaCl), 0.5% blocking buffer (CAT# 1152709001: Roche). Antibodies were diluted as follows: rat anti-AKMT 1∶500, mouse anti-tubulin B-5-1-2 1∶2,000 (CAT# T6074: Sigma), mouse anti-actin 1∶5,000 (CAT# MAB1501: Millipore), rabbit anti-TgMIC2 1∶5,000, rabbit anti-TgPLP1 1∶1,500 (anti-TgMIC2 and anti-PLP1 antibodies were kind gifts from Dr. Vern Carruthers, University of Michigan), goat anti-rat IgG HRP 1∶2,000 (CAT# NA935V: GE Healthcare) and goat anti-mouse/rabbit HRP 1∶20,000 (CAT# 1152709001: Roche).

### Protein lysine methyltransferase (PKMT) assay

PKMT assay was performed as described [68], [69] with some modifications. For activity assays with recombinant wild-type and mutant AKMT proteins (FLAG-AKMT and FLAG-akmt(H447V), respectively), 30 µl reactions were set up with varying amounts of FLAG-AKMT (0.01, 0.05, 0.1, 0.5, 1 µg) or FLAG-akmt(H447V) (1 µg) proteins, 1 µg Xenopus histone H3.3 (CAT# 14-411: Millipore), 0.5 mM DTT, 2 µCi ^3^H-S-adenosyl-L-methionine (^3^H-SAM) (CAT# NET155V250UC: Perkin Elmer) in “intracellular (IC) buffer” [51] or DPBS. For tandem mass spectrometry of Xenopus histone H3.3, 22 µl reactions were set up with 1 µg FLAG-AKMT protein, 1 µg histone, 0.5 mM DTT, and 0.9 µM S-adenosyl-L-methionine (SAM) (CAT# 7007: Sigma) in DPBS. Reactions were incubated at 30°C for 60 minutes, and then stopped by adding LDS sample buffer and reducing agent and 10 min 70°C heating. Proteins in the reactions were resolved on 4–12% gradient bis-tris NuPAGE gels (CAT# NP0332: Invitrogen). For identification of peptides containing methylated lysine residues, histone H3.3 bands were excised from the gel and analyzed by tandem mass spectrometry (National Center for Glycomics & Glycoproteomics, Indiana University). For detecting the methylated histone by autoradiography, proteins resolved on NuPAGE gels were transferred to PVDF. To visualize total proteins, the PVDF membranes were first washed twice in DPBS for 5 minutes each, stained with amido black staining solution (0.1% amido black 10B, 45% methanol, 10% acetic acid) for 5–10 min and rinsed briefly 4–6 times in destaining solution (90% methanol and 2% acetic acid). The membranes were then air-dried, sprayed two or three times with EN^3^HANCE Spray (CAT# 6NE970C: Perkin Elmer) as per manufacturer's instructions and exposed to X-ray film for 24–72 hours as necessary to detect the ^3^H signal incorporated into the histone H3.3 protein.

### Plaque assay

Plaque assays were performed as previously described [48]. 1,000 or 10,0000 parasites were added to T12.5 tissue culture flasks and grown undisturbed for 7 or 14 days.

### Immunofluorescence

3D image stacks were collected at z-increments of 0.3 µm using an Applied Precision Delta Vision imaging station constructed on an Olympus inverted microscope base. A 100X oil immersion lens (1.4 NA), and immersion oil of refractive index 1.518 or 1.524 was used for the imaging at room temperature or 37°C respectively. Deconvolved images were computed using the point-spread functions and software supplied by the manufacturer. The brightness and contrast of images used in the final figures were optimized for color prints. For immunolabeling, unless otherwise noted, parasites grown in HFF monolayer were fixed in 3.7% formaldehyde in DPBS for 15 minutes and permeabilized with 0.25% TX-100 in DPBS for 15 minutes at room temperature. Cells were blocked with 1% BSA in DPBS for 5 to 30 minutes at room temperature and then incubated in primary and subsequently secondary antibody solutions for 30 minutes each. Primary antibody dilutions were as follows: mouse anti-IMC1, 1∶1000 (A kind gift from Dr. Gary Ward, University of Vermont); affinity purified rat anti-AKMT, 1∶50; mouse anti-SAG1 (CAT# 11–132: Argene), 1∶1000. Goat anti-mouse Alexa 568 (CAT# A11031: Molecular Probes-Invitrogen); goat anti-mouse Alexa488 (CAT# A11031: Invitrogen); goat anti-rat Alexa 488 (CAT# A11006: Molecular Probes-Invitrogen); and goat anti-rat Cy5 (CAT# A 21236: Molecular Probes-Invitrogen) were used at 1∶1000–2000 dilution. Coverslips were mounted on slides with ProLong Gold antifade (CAT# 36930: Invitrogen).

### Intracellular replication assay

Intracellular replication assay was performed as previously described [48]. Parasites were grown for 12, 24 or 36 hours. The number of parasites/vacuole was determined in 100 vacuoles at each time point in three independent experiments and the replication rate was determined as previously described [70].

### Invasion assay

Invasion assay was performed as previously described [49], [50] with slight modifications. Briefly, for each sample, 1×10^7^ parasites from a freshly lysed culture were added to one coverslip in a six well plate containing confluent HFF monolayers and incubated at 37°C for 1 hour, washed with DPBS. Cells were fixed with 3.7% formaldehyde for 15 minutes, blocked with 1% BSA in DPBS (BSA/DPBS) for 5 minutes and incubated in mouse anti-SAG1 diluted 1∶1000 for 1 hour and then in goat anti-mouse Alexa 568 diluted 1∶1000 for 1 hour. Cells were permeabilized in 0.25% TX-100 in DPBS for 15 minutes, blocked with BSA/DPBS for 5 minutes and incubated in rabbit anti-IMC1 (a kind gift from Dr. Con Beckers, University of North Carolina, Chapel Hill) diluted 1∶500 for 1 hour and then in goat anti-rabbit Alexa 488 (CAT# A11034: Invitrogen) diluted 1∶1000 for 1 hour. The samples were then imaged using an Olympus 10X UPLanSApo objective (N.A. = 0.4). To count invaded parasites (i.e. parasites that were labeled with anti-IMC1/goat-anti-rabbit Alexa488 but not labeled with anti-SAG1/goat-anti-mouse Alexa568), a binary mask was first created in Metamorph (Molecular Devices) using the SAG1-Alexa568 channel, where all extracellular parasites were brightly labeled. The mask was then subtracted from the IMC1-Alexa488 channel to exclude all extracellular parasites. The remaining positively labeled parasites in the IMC1-Alexa488 channel were then counted for each field. For each experiment, 10 fields were counted for every sample. Three independent experiments were performed.

### Egress assays

#### Immunofluorescence-based induced-egress assays

3×10^3^ (for *RHΔhx*, *loxp_akmt knockin,* and *flag-akmt complements*) or 4×10^4^ (for *Δakmt* parasites) parasites were added to each well of a 96 well glass-bottom plate (CAT# MGB096-1-2-HG: MatriCal) containing confluent HFF monolayers and grown for ∼36 hours. Cells were washed once and incubated in CO_2_ independent media (Sku# RR060058: Invitrogen) pre-warmed to 37°C containing either 5 µM A23187 (from a stock solution of 10 mM in DMSO) or an equivalent volume of DMSO alone for 0, 2, 5 and 10 minutes. Cells were fixed by the addition of an equal volume of 2X fixative solution (6% formaldehyde) at room temperature for 15 minutes. Cells were then blocked with 1% BSA/DPBS for 5 minutes and incubated in mouse anti-SAG1 diluted 1∶1000 for 15 minutes and then in goat anti-mouse Alexa 568 diluted 1∶500 for 15 minutes to label parasites that were accessible to the antibodies (*i.e.* extracellular parasites or parasites contained within permeabilized vacuoles and host cells). Cells were permeabilized in 0.25% TX-100 in DPBS for 15 minutes, blocked with 1% BSA/DPBS for 5 minutes and incubated in rabbit-anti GAP45 or IMC1 (kind gifts from Dr. Con Beckers, University of North Carolina, Chapel Hill) diluted 1∶1000 for 15 minutes and then in goat anti-rabbit Alexa 488 diluted 1∶1000 for 15 minutes to label all parasites. 50 vacuoles/time point were scored and classified as 1) egressed if parasites had dispersed from the vacuole; 2) permeabilized if parasites were retained in the parasitophorous vacuole but some or all of the parasites in the vacuole were positively labeled with anti-SAG1; 3) intact if none of the parasites in the vacuole were positively labeled with anti-SAG1.

#### Live cell imaging/analysis of egress

For natural egress assay, parasites were added to MatTek dishes (CAT# P35G-1.5-14-C: MatTek Corporation) containing confluent HFF monolayers and grown for ∼40 hours. Cells incubated in 3–4 ml of CO_2_ independent media containing 1% heat-inactivated bovine calf serum (CO_2_ independent imaging media) were imaged every 30–90 seconds for 5–6 hours.

For induced egress assay, parasites were added to MatTek dishes containing a confluent HFF monolayer and grown for ∼35–40 hours. Cells were washed once and incubated in 1 or 1.5 ml of CO_2_ independent imaging media. 1 ml of 10 µM A23187 or 0.5 ml of 20 µM A23187 in CO_2_ independent imaging media was then added to the MatTek dishes to induce egress. Images were collected at 37°C on an Applied Precision Delta Vision imaging station equipped with an environmental chamber.

For assessing the functionality of GFP-AKMT(WT) and GFP-akmt(H447V), *Δakmt* parasites were harvested, transfected with pmin-eGFP-AKMT(WT) or pmin-eGFP-akmt(H447V), and inoculated on MatTek dishes. After 36–40 hours, parasites expressing GFP-AKMT alleles were treated with 5 µM A23187 as described above and induced egress was observed for 20–30 minutes afterwards.

To quantify the eGFP-AKMT dissociation from the apical complex over time upon A23187 treatment (Figure 8A), images were collected as follows. After imaging two time-points 30 seconds apart, 1 ml of 10 µM A23187 in CO_2_ independent imaging media was added to the MatTek dishes containing 1 ml of CO_2_ independent imaging media and images were collected every 6 seconds afterwards. Six planes were collected at 0.5 µm intervals for each time-point. Summed projections of the fluorescence 3-D stacks were then generated. The average fluorescence intensity of seven mother apical complexes was calculated in Metamorph. The average fluorescence intensity of the apical complex (background subtracted) was then divided by the average fluorescence intensity of the entire vacuole (background subtracted) to give the bleach-corrected relative fluorescence intensity of the mother apical complex.

To assess the effect of actinomycin D (Cat#A9415: Sigma) and cycloheximide (Cat#C7698: Sigma) treatment on parasite motility activation for egress, *RHΔhx* parasites were added to MatTek dishes containing a confluent HFF monolayer and grown for ∼20–26 hours in DMEM with 1% BCS at 37°C with 5% CO_2._ The cultures were then treated with 0.5 µg/ml actinomycin D or 10 µM cycloheximide in DMEM with 1% BCS for various periods of time at 37°C with 5% CO_2_. Before imaging, the media was replaced by 1.5 ml of CO_2_ independent imaging media containing 0.5 µg/ml actinomycin D or 10 µM cycloheximide. 0.5 ml of 20 µM A23187 in CO_2_ independent imaging media containing 0.5 µg/ml actinomycin D or 10 µM cycloheximide was then added to the MatTek dishes and induced egress was observed for 5–10 minutes afterwards.

### Apical complex extension assay

Assays were performed as previously described [9], [50]. Apical complex extension was induced with 5 µM A23187 in DPBS containing 0.9 mM calcium and 0.49 mM magnesium (CAT# 14040−133: Invitrogen). Control samples were treated with an equivalent volume of DMSO. DIC images were taken by a 100X Olympus objective (N.A. = 1.4). 300 parasites/slide were scored for apical complex extension in each of three independent experiments.

### Trail (motility) assay

The assay was performed as previously described [17]. Trails were visualized with mouse anti-SAG1 diluted 1∶100 and goat anti-mouse Alexa488 diluted 1∶500.

### The characterization of AKMT distribution in potassium and sodium based buffers

#### Localization of eGFP-AKMT in “intracellular (IC) buffer” and “extracellular (EC) buffer.”

Extracellular *egfp-akmt complement* parasites were washed once and resuspended and incubated in either potassium based “intracellular (IC) buffer” (120 mM KCl; 0.15 mM CaCl^2^; 10 mM KH^2^PO^4^/K^2^HPO^4^ pH 7.6; 25 mM HEPES-KOH pH 7.6; 2 mM EGTA pH 7.6; 5 mM MgCl^2^, pH adjusted with KOH) [51] or sodium based “extracellular (EC) buffer” [8] (120 mM NaCl; 0.15 mM CaCl^2^; 10 mM NaH^2^PO^4^/Na2HPO4 pH 7.6; 25 mM HEPES-NaOH pH 7.6; 2 mM EGTA pH 7.6; 5 mM MgCl2, pH adjusted with NaOH) for 20–30 minutes. Parasite suspensions were then placed on a glass slide, covered with coverglass (#1.5), and imaged using an Applied Precision Delta Vision imaging station and a 100X oil immersion lens (1.4 NA). Line scans were generated in Metamorph (Molecular Devices).

#### Localization of eGFP-AKMT in buffer exchange experiments

Freshly lysed extracellular *egfp-akmt complement* parasites were first incubated in EC or IC buffer for 25–30 minutes, spun for 5 minutes at 6000 rpm, and then resuspended in buffer containing different ionic composition or drugs that affect intra-parasite [Ca^2+^] concentration. We observed the localization of AKMT before and after the buffer exchange by taking an aliquot of the parasites from the suspension (kept at 37°C) every 3–4 minutes and preparing the sample for imaging as described above. A 60X water objective and 2×2 binning was used for imaging. The earliest time-point that we could image AKMT localization after resuspending the parasites in a new buffer was ∼2.5 to 3 minutes after the buffer exchange.

### Microneme secretion assay

The assay was performed as previously described [15] except that microneme secretion was induced for 30 minutes by A23187 treatment. Western blot was performed as described above with anti-TgMIC2 and anti-TgPLP1 antibodies [16] (kind gifts from Dr. Vern Carruthers, University of Michigan). Actin was used as a loading control.

### Scanning electron microscopy

10 µl of a suspension containing *Δakmt* parasites was placed on a 12 mm coverslip that had been coated with 1% poly L-lysine (CAT# P-1274: Sigma). After 5 minutes excess parasite suspension was removed with filter paper. 1% glutaraldehyde in 25 mM sodium phosphate buffer, pH 6.2 was then placed on the coverslip. After 30 minutes the coverslip was rinsed with 25 mM sodium phosphate buffer, pH 6.2; covered with 1% osmium tetroxide in 25 mM sodium phosphate buffer, pH 6.2 for 1 hour; and then rinsed with the sodium phosphate buffer and dehydrated in a graded ethanol series. All the steps from fixation to the initial dehydration steps were done while the coverslip was on ice. The final dehydration step with 100% ethanol was done at room temperature. The coverslip was critical point dried with a Balzers CPD030, sputter coated with a Polaron E5100 with a palladium (80%)/gold (20%) target, and viewed with a JEOL 5800 LV SEM at 15 kV.

### Uracil [5,6-^3^H] labeling of intracellular parasite RNA

Freshly lysed *RHΔhx* parasites were inoculated on HFF in T12.5 flasks in DMEM with 1% BCS. After overnight to 24 hr incubation at 37°C with 5% CO_2_, flasks were washed with media to remove any extracellular parasites. Actinomycin D (Cat#A9415: Sigma; 1 mg/ml stock solution in DMSO) was added to infected flasks to the final concentration of 0.5 µg/ml. After 5.5 hr incubation at 37°C, 5 or 20 µCi/ml uracil [5,6-^3^H] (Cat#NET368250UC: Perkin Elmer) was added per flask and incubated for 2 hr to label newly synthesized RNA. To estimate the uracil [5,6-^3^H] labeling of intracellular parasite RNA in the absence of actinomycin D, equivalent volume of DMSO was used instead of actinomycin D. For each sample, 5×10^6^ intracellular parasites were harvested after needle-pass and filtration and washed once in IC buffer (see above, also [8], [51]) with or without actinomycin D. Total RNA was extracted using RNeasy kit (Cat#74104: Qiagen) with on-column DNase treatment (Cat#79254: Qiagen). 1/5 of the extracted RNA was used to measure ^3^H labeling by liquid scintillation counting. Two independent experiments, each with two replicates, were performed.

### ^35^S labeling of intracellular parasite proteins

Assay was performed as described in [58] with some minor modifications. Specifically, freshly lysed *RHΔhx* parasites were inoculated on HFF in T12.5 flasks in DMEM with 1% BCS. After overnight to 24 hr incubation at 37°C with 5% CO_2_, flasks were washed three times with cysteine and methionine-free DMEM media with 3% dialyzed FBS (Cat#26400-036: Invitrogen), and incubated for 30 min. To inhibit cytoplasmic protein synthesis, intracellular parasites were incubated with cycloheximide (Cat#C7698: Sigma; 10 mg/ml stock solution in dH_2_O) at the final concentration of 10 µM for 2 hr, and 30 µCi of EasyTag EXPRESS^35^S Protein Labeling Mix, [^35^S] (Cat#NEG772007MC: Perkin Elmer) was added per flask and incubated for 1 hr to label newly synthesized proteins. As the positive control for ^35^S labeling, parasites were incubated with the protein labeling mix without cycloheximide. For each sample, 5×10^6^ intracellular parasites were harvested after needle-pass and filtration, washed once in IC buffer with or without cycloheximide, and lysed in 100 µl 0.05% sodium deoxycholate in IC buffer with or without cycloheximide. After the addition of 25 µl of 10N NaOH, lysates were incubated at 37°C for 15 min to hydrolyze nucleic acids. 1/5 volume of lysates were precipitated by TCA precipitation and spotted on the glass GF/C filters (Cat#1822024: Whatman) on vacuum manifold. Filters were washed with 5% TCA followed by ethanol, and dried. ^35^S labeling on filters was measured using liquid scintillation counting. Two independent experiments, each with two replicates, were performed.

### Accession numbers

TgAKMT (TGME49_016080; **EupathDB**);

NcAKMT (NC_LIV_060040; **EupathDB**);

PfAKMT (PF11_0160; **EupathDB**);

CpAKMT (cgd4_2090; **EupathDB**)

BbAKMT (XP 00611004.1; **GenBank**)

TaAKMT (XP 954031.1; **GenBank**) TgMORN1 (583.m05359, **EupathDB**)

TgIMC1 (44.m00004, **EupathDB)**

TgGAP45 (AF453384.1, **GenBank**)

TgSAG1 (TGME49_033460, **EupathDB**)

TgTubulinA1 (583.m00022, **EupathDB**)

## Supporting Information

Figure S1 **Characterization of the enzymatic domain and the localization of AKMT.** (A) Alignment of the SET and zinc binding domain of AKMT orthologs found in several apicomplexan parasites. Accession number of these orthologs are: TGME49_016080 (*T. gondii*; EupathDB); NC_LIV_060040 (*N. caninum;* EupathDB); PF11_0160 (*P. falciparum*; EupathDB); cgd4_2090 (*C. parvum*; EupathDB); XP 00611004.1(*B. bovis*; GenBank); XP 954031.1(*T. annulata*; GenBank). Conserved amino acids in each motif found in canonical SET domains are shown above the *T. gondii* AKMT sequence. (B) Protein sequence of Xenopus histone H3.3. Peptides containing lysine methylated by AKMT in *in vitro* PKMT assay are highlighted in red. (C) AKMT localization in intracellular parasites at different stages of parasite replication. *Top*: Two parasites at the beginning of daughter construction where the daughter apical (small yellow arrows) and basal complexes (small arrowheads) are being constructed around the parasite centriole/spindle pole assembly (white arrows) [43] (*T. gondii* replicates by forming daughters in the mother, therefore the daughters have their own sets of apical and basal complexes.). AKMT had already been recruited to the daughter apical complex at this stage (Inset). Inset 2X magnification. *Bottom*: Two dividing parasites in which the daughter apical and basal complexes had separated from each other due to the growth of the daughter cortical cytoskeleton. *Green:* anti-AKMT; *red*: mCherryFP-TubulinA1 [43], [44], highlighting all the tubulin containing structure in the parasite, including the main body of the cytoskeletal apical complex (yellow arrows) of both the mother and the daughter (M-AC: mother apical complex; D-AC: daughter apical complex) as well as the centriole/spindle pole assembly (white arrows); *cyan*: eGFP-MORN1 [32], [43], [45], highlighting the basal complex (arrowheads) of both the mother and the daughter (M-BC: mother basal complex; D-BC: daughter basal complex) as well as the spindle pole (white arrows).
(PDF)

Figure S2 **Genomic PCR and Western blot analyses of the** ***RHΔhx*** **,** ***loxp_akmt knockin*** **,** ***Δakmt*** **and** ***flag-akmt complement*** **parasites.** (A) *Top*: Diagram of the genomic locus of *loxp_akmt knockin* parasites. Annealing sites for primers (S1, A1; S2, A2) used for the PCR analysis are indicated in the diagram. *Bottom*: Genomic PCR analysis of the *RHΔhx*, *loxp_akmt knockin*, *Δakmt* and *flag-akmt complement* parasites. (B) *Top*: Western blot analysis of the *RHΔhx*, *loxp_akmt knockin*, *Δakmt* and *flag-akmt complement* parasites using rat anti-AKMT antibody, showing that AKMT was undetectable in the *Δakmt* parasite lines. *Bottom*: reprobing of the blot above with mouse anti-tubulin B-5-1-2 to use alpha-tubulin as a loading control.
(PDF)

Table S1 **Primers used for PCR amplification in plasmid construction.** Primers used for PCR amplification in the construction of the plasmids listed in the right column. Restriction sites are shown in lower case.
(DOC)

Video S1 **Live video microscopy of A23187 induced-egress for** ***loxp_akmt knockin*** **,** ***Δakmt*** **, and** ***flag-akmt complement*** **parasites.** Live video microscopy of A23187 induced-egress for *loxp_akmt knockin* (left), *Δakmt* (middle), and *flag-akmt complement* parasites (right) grown in HFF. A23187 was added at the beginning of the movies to the final concentration of 5 µM. The time interval between each frame is 1 second. Bar = 15 µm.
(MOV)

Video S2 **Live video microscopy of natural egress for** ***loxp_akmt knockin*** **,** ***Δakmt*** **, and** ***flag-akmt complement*** **parasites.** Live video microscopy of natural egress for *loxp_akmt knockin* (left), *Δakmt* (middle), and *flag-akmt complement* parasites (right) grown in HFF. The time interval between each frame is 90 seconds. To display the egress process in synchrony, the time point at which parasite egress (for *loxp_akmt knockin* and *flag-akmt complement* parasites) or host cell permeabilization (for *Δakmt* parasites) occurs is chosen as the 0∶00∶00 time point for all videos. Notice that for *loxp_akmt knockin* and *flag-akmt complement* parasites, host cell permeabilization and parasite egress occur almost simultaneously and many parasites invade adjacent host cells immediately after egress. *Δakmt* parasites however, remain largely immotile and fail to exit the host cell actively. Bar = 15 µm.
(MOV)

Video S3 **Live video microscopy of A23187 induced-egress for intracellular** ***Δakmt*** **parasites expressing eGFP-AKMT(WT).** Live video microscopy of A23187 induced-egress for intracellular *Δakmt* parasites expressing eGFP-AKMT(WT). A23187 was added between 0∶00∶30 and 0∶00∶49 to the final concentration of 5 µM. The time interval between each frame is 6 seconds.
(MOV)
