# Supplementary material for: The Motility of a Human Parasite, Toxoplasma gondii, Is Regulated by a Novel Lysine Methyltransferase
Source: PLoS Pathog. 2011 Sep 1;7(9):e1002201. doi: 10.1371/journal.ppat.1002201 (PMC3164638; doi:10.1371/journal.ppat.1002201)
Supplement: Figure S1 — Characterization of the enzymatic domain and the localization of AKMT. (A) Alignment of the SET and zinc binding domain of AKMT orthologs found in several apicomplexan parasites. Accession number of these orthologs are: TGME49_016080 (T. gondii; EupathDB); NC_LIV_060040 (N. caninum; EupathDB); PF11_0160 (P. falciparum; EupathDB); cgd4_2090 (C. parvum; EupathDB); XP 00611004.1(B. bovis; GenBank); XP 954031.1(T. annulata; GenBank). Conserved amino acids in each motif found in canonical SET domains are shown above the T. gondii AKMT sequence. (B) Protein sequence of Xenopus histone H3.3. Peptides containing lysine methylated by AKMT in in vitro PKMT assay are highlighted in red. (C) AKMT localization in intracellular parasites at different stages of parasite replication. Top: Two parasites at the beginning of daughter construction where the daughter apical (small yellow arrows) and basal complexes (small arrowheads) are being constructed around the parasite centriole/spindle pole assembly (white arrows) [43] (T. gondii replicates by forming daughters in the mother, therefore the daughters have their own sets of apical and basal complexes.). AKMT had already been recruited to the daughter apical complex at this stage (Inset). Inset 2X magnification. Bottom: Two dividing parasites in which the daughter apical and basal complexes had separated from each other due to the growth of the daughter cortical cytoskeleton. Green: anti-AKMT; red: mCherryFP-TubulinA1 [43], [44], highlighting all the tubulin containing structure in the parasite, including the main body of the cytoskeletal apical complex (yellow arrows) of both the mother and the daughter (M-AC: mother apical complex; D-AC: daughter apical complex) as well as the centriole/spindle pole assembly (white arrows); cyan: eGFP-MORN1 [32], [43], [45], highlighting the basal complex (arrowheads) of both the mother and the daughter (M-BC: mother basal complex; D-BC: daughter basal complex) as well as the spindle pole (white a [file ppat.1002201.s001.pdf]

A

```
                          GXG
T.gondii         ATKVQVKHVPGKGRCLYTKHDLEPGSIIFVETPVLVAIPSLDEELWSVLTEINDEEALEL
N.caninum        ATKVQVRHVPGKGRCLYTKHDLEPGAIIFVETPVLVAIPSLDEELWSALTEINDEEALEL
P.falciparum     ---VEIFNVKGKGRCMFTKKKLDPGSVIFVENPILIVTPNLNEQLWTYLNKLNDEQNFEL
C.parvum         HESVEVRYTESKGRCLYARKCFNPGDIIFAESPLLVVTPELAPELSEFLEDMNSKETFTL
B.bovis          LSLVEIAPAPGKGRCMYTRRAYEPGEIIIIEKPLFAITPDSNSNIWDTLMTLHQEQPLHL
T.annulata       -SLVRVSEVPGKGRCLFVRKAFDPGELIFAESPLFMIDPTSNQELWDELNKLNEESPFVL
                     *.:   . .****::.::  :** :*: *.*::    *     ::    *  ::.:. : *
                         Motif I

T.gondii         PPVWHLAAICSLTMLDDEKKKICLDKWVPDPDRAPSDDVLRVINRAGLQ-----------
N.caninum        PPVWHLAAICSLTMLDDEKKQICLDKWVPDPDRPPSDDVLRVINRAGLE-----------
P.falciparum     PLKWHYAALCSITMLNDFNYKACLDKWVPEPDKEPDNDIYNVLDKVCEKTSFVNGNKYYY
C.parvum         PPLWHVAALCTLTMLEEEDKAICLDKWVPDPSAEPSYDVQKIIENTGID-----------
B.bovis          PPLWHHAALVSILEGNEEKLEIMKGKWVLDNDPPVSEDVYRILEVTCAEHR---DGSFVY
T.annulata       PPLWHKAALFSILEGNEESNSILENKWVENRNQEVSADVLRVLNSICTIE----DGEFYY
                 *  ** **: ::   :: .      .*** : .    . *: .:::

                                                     RFINHxCxPN
T.gondii         -----VHPKLYERMLMVWRYNSFGHHTEQHGLVLYNRISMMAHSCRATACWHYGEDDAFI
N.caninum        -----VHPQLYERMLMVWRYNSFGHHTEQQGLVLYNRISMMAHSCRATACWHYGEDDAFV
P.falciparum     YKNKLIDPKIYSRIIQVWHYNAFGHHTDNEGLVLYNRISMLAHSCISTACWHYGENDSFV
C.parvum         -----VDPHLYERTLNAWRFNSFN-HTSNNGIVLYNVISMMAHNCGASCCWHYGVDNTFV
B.bovis          HNGTVICPNLYQFLLQVWPLNAFAHSTHPQGLVIYDKISYLAHSCDSSATWNHYGEDIFV
T.annulata       D-GVVIKPEVYQLYLQVWPLNAFGRTSEPDGLVIYDKISYVAHSCNPSCCWHHTENDEFV
                      : *.:*.  : .*  *:*   :  .*:*:*: ** :**.* .:. *::  :: *:
                                                         Motif III
                              ELxFDY
T.gondii         LRARVKLQAGDELTISYIGDDDLFKSTNVRREKVYGWLFTCQCVRCAAPVDNARGFRCPL
N.caninum        LRARVKLQAGDELTISYIGDDDLFKSTNVRREKVQGWLFTCQCVRCAAPVDNARGFRCPL
P.falciparum     LRARINLNPGDEITISYLGDDDLYKSSNIRREKLTNWLFVCMCSRCTHPVDNCRGFRCSS
C.parvum         LRAKTRLEVGDEITISYISDDDLFKCSKTRRELLSNWLFYCQCERCNNPTDLSRGLKCAS
B.bovis          LRARRKLQIGDELTISYIGEADLLAPTP------SKWD----------------------
T.annulata       LRARKKLVPGDEITISYLGETDLLSPTFRRRTLLQNWHFFCTCERCSIPIDNCRGFLCKC
                 ***: .*  ***:****:.: **   :          *   Zn-
                              Motif IV                   binding

T.gondii         CGTGAMFF
N.caninum        CGTGAMFF
P.falciparum     CGIGTFF-
C.parvum         CGVGSMFF
B.bovis          --------
T.annulata       CHYGTIF-
```

B

MARTKQTARKSTGGKAPRKQLATKAARKSAPSTGGVKKPHRYRPGTVALREIRRYQKSTELLIRKLPFQRL
VREIAQDFKTDLRFQSAAIGALQEASEAYLVGLFEDTNLCAIHAKRVTIMPKDIQLARRIRGERA

C

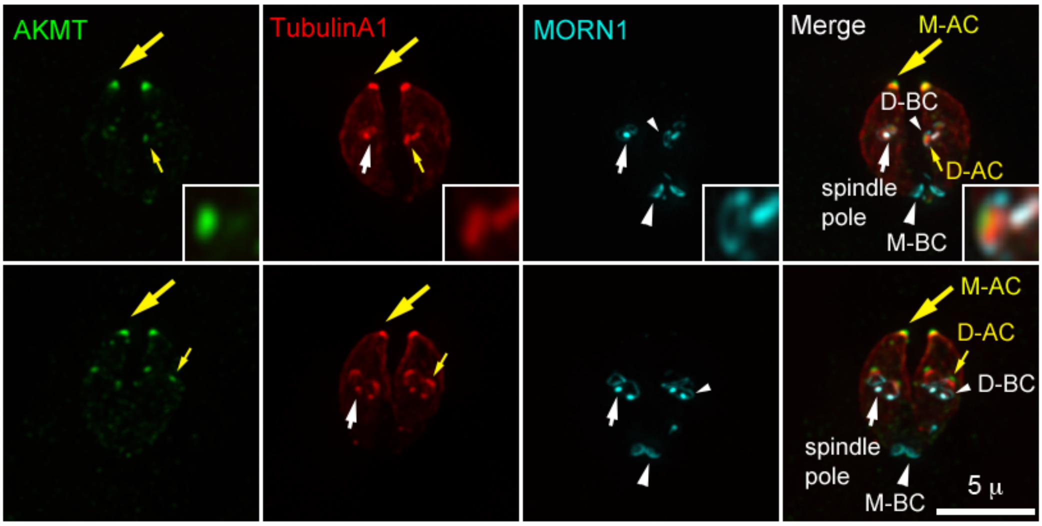

Figure S1
