# Supplementary figures and images for: The Motility of a Human Parasite, Toxoplasma gondii, Is Regulated by a Novel Lysine Methyltransferase
Source: PLoS Pathog. 2011 Sep 1;7(9):e1002201. doi: 10.1371/journal.ppat.1002201 (PMC3164638; doi:10.1371/journal.ppat.1002201)

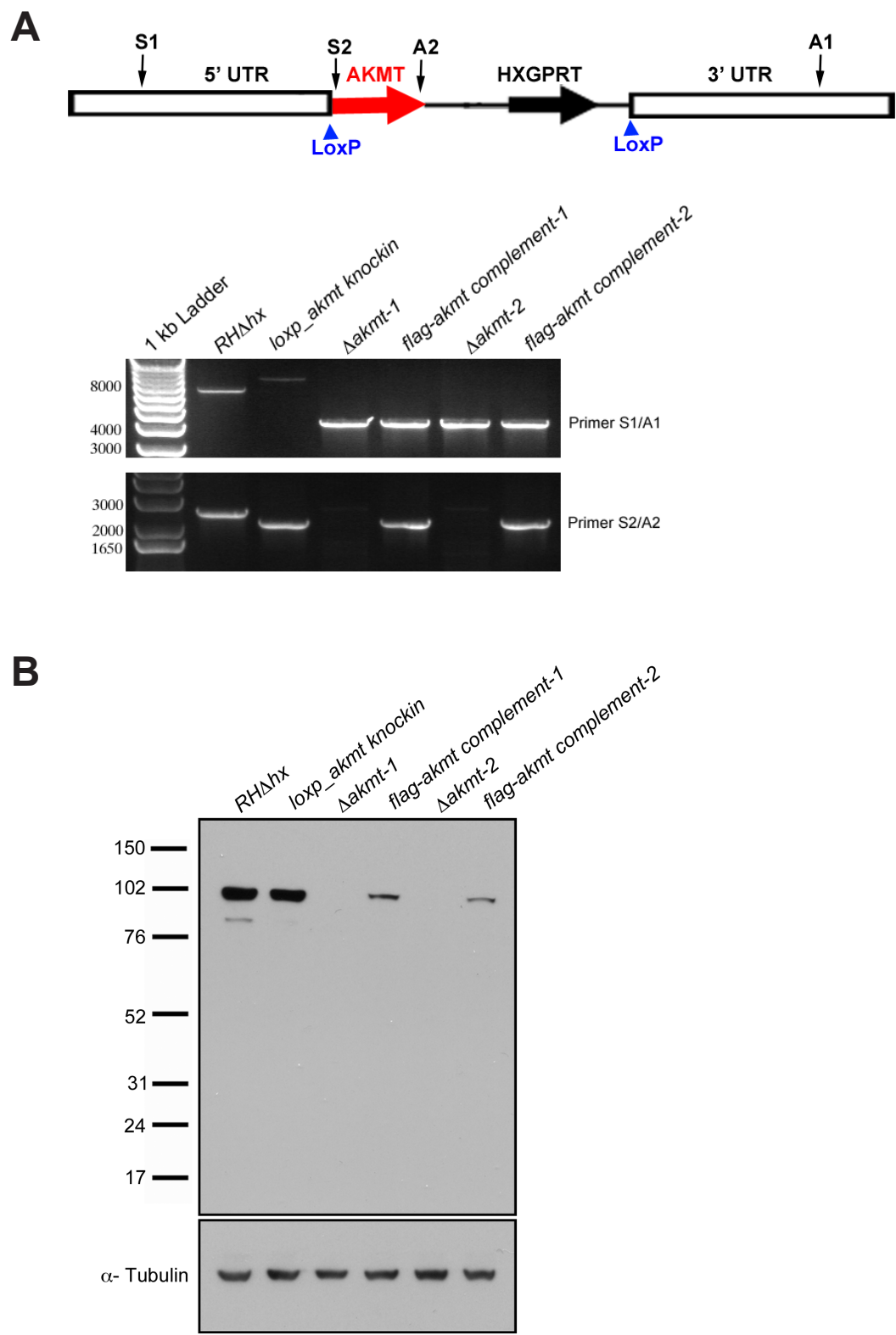

Figure S2

Supplement: Figure S2 — Genomic PCR and Western blot analyses of the RHΔhx , loxp_akmt knockin , Δakmt and flag-akmt complement parasites. (A) Top: Diagram of the genomic locus of loxp_akmt knockin parasites. Annealing sites for primers (S1, A1; S2, A2) used for the PCR analysis are indicated in the diagram. Bottom: Genomic PCR analysis of the RHΔhx, loxp_akmt knockin, Δakmt and flag-akmt complement parasites. (B) Top: Western blot analysis of the RHΔhx, loxp_akmt knockin, Δakmt and flag-akmt complement parasites using rat anti-AKMT antibody, showing that AKMT was undetectable in the Δakmt parasite lines. Bottom: reprobing of the blot above with mouse anti-tubulin B-5-1-2 to use alpha-tubulin as a loading control. (PDF) [file ppat.1002201.s002.pdf]
