# Supplementary material for: The Motility of a Human Parasite, Toxoplasma gondii, Is Regulated by a Novel Lysine Methyltransferase
Source: PLoS Pathog. 2011 Sep 1;7(9):e1002201. doi: 10.1371/journal.ppat.1002201 (PMC3164638; doi:10.1371/journal.ppat.1002201)
Supplement: Table S1 — Primers used for PCR amplification in plasmid construction. Primers used for PCR amplification in the construction of the plasmids listed in the right column. Restriction sites are shown in lower case. (DOC) [file ppat.1002201.s003.doc]

**TABLE S1.** Primers used for PCR amplification in the construction of the plasmids listed in the right column. Restriction sites are shown in lower case.

| Primer Name: Sequence | Used for the construction of: |
| --- | --- |
| S112: ATGCagatctAGCCGTCGCGTTTCCCACAACAGTTC  A113: ATCGcttaagTCAACTGGCCGGTAGGCGTTCCTCG | pmin-eGFP-AKMT |
| AKMT-5’UTR-S1: AGTgcggccgcCGACATTCTGCTCATAGTGTCCGT  AKMT-5’UTR-A1: AGTgaattcCCACGAGCGATCTGTTAGATTAAG | pTKO2_II_AKMT_CKO (amplification of AKMT 5’ UTR) |
| AKMT-3’UTR-S1: AGTgctagcAGTTTGAGCACCCTTTAGAGTCTGA  AKMT-3’UTR-A1: AGTgggcccAGGCGCCGCTGCTGCAGAAAGCTTA | pTKO2_II_AKMT_CKO (amplification of AKMT 3’ UTR) |
| AKMT-CDS-S1: AGTgtttaaacTTCTGACAAAATGAGCCGTCGCGTTTCCCACAACA (Underlined: Kozak sequence)  AKMT-CDS-A1: AGTcggaccgTCAACTGGCCGGTAGGCGTTCCTCGCA | pTKO2_II_AKMT_CKO (amplification of AKMT coding sequence) |
| AKMT-301F : ATGCagatctGCTACCAAGGTGCAGGTCAAGCA  A113: ATCGcttaagTCAACTGGCCGGTAGGCGTTCCTCG | pmin-eGFP-AKMT 301-709aa (amplification of AKMT 301-709aa) |
| NheI-AKMT-S: ATGCgctagcAGCCGTCGCGTTTCCCACAACAGTTC  EcoRI-AKMT-A: ATCGgaattcTCAACTGGCCGGTAGGCGTTCCTCG | pET22b-FLAG -AKMT (amplification of AKMT CDS) |
| NheI-AKMT-S: ATGCgctagcAGCCGTCGCGTTTCCCACAACAGTTC  M13 reverse: CAGGAAACAGCTATGAC  AKMT-301F: ATGCagatctGCTACCAAGGTGCAGGTCAAGCA  EcoRI-AKMT-A: ATCGgaattcTCAACTGGCCGGTAGGCGTTCCTCG | pET22b-FLAG-akmt(H447V) (amplification of akmt(H447V) CDS) |
